# Genomes on a Tree (GoaT): A versatile, scalable search engine for genomic and sequencing project metadata across the eukaryotic tree of life

**DOI:** 10.12688/wellcomeopenres.18658.1

**Published:** 2023-01-17

**Authors:** Richard Challis, Sujai Kumar, Cibele Sotero-Caio, Max Brown, Mark Blaxter

**Affiliations:** 1 Tree of Life, Wellcome Trust Sanger Institute, Cambridge, CB10 1SA, UK

**Keywords:** Genomics; Earth BioGenome Project; Tree of Life; Databasing; Elasticsearch

## Abstract

As genomic data transform our understanding of biodiversity, the Earth BioGenome Project (EBP) has set a goal of generating reference quality genome assemblies for all ~1.9 million described eukaryotic taxa. Meeting this goal requires coordination among many individual regional and taxon-focussed projects working under the EBP umbrella. Large-scale sequencing projects require ready access to validated genome-relevant metadata, such as genome sizes and karyotypes, but these data are dispersed across the literature, and directly measured values are lacking for most taxa. To meet these needs, we have developed Genomes on a Tree (GoaT), an Elasticsearch-powered datastore and search index for genome-relevant metadata and sequencing project plans and statuses.

GoaT indexes publicly available metadata for all eukaryotic species and interpolates missing values through phylogenetic comparison. GoaT also holds target priority and sequencing status information for many projects affiliated to the EBP to aid project coordination. Metadata and status attributes in GoaT can be queried through a mature API, a web front end, and a command line interface. The web front end additionally provides summary visualisations for data exploration and reporting (see https://goat.genomehubs.org).

GoaT currently holds direct or estimated values for over 70 taxon attributes and over 30 assembly attributes across 1.5 million eukaryotic species.

The depth and breadth of curated data, frequent updates, and a versatile query interface make GoaT a powerful data aggregator and portal to explore and report underlying data for the eukaryotic tree of life. We illustrate this utility through a series of use cases from planning through to completion of a genome-sequencing project.

## Introduction

The Earth BioGenome Project (EBP) aims to sequence the complete genomes of every one of the ~1.9 million described eukaryotic species over the next 10 years ^1^. This challenge is being met by an engaged community of individual EBP partner projects with lists of target species defined by geographic region, taxonomic clade, or based on criteria of economic importance, conservation status, or ecosystem service. The nature of species distributions coupled with the diverse definitions of these partner projects results in overlapping target species lists. Coordination of sequencing efforts across the different projects is essential to prevent duplication, to focus efforts on underrepresented branches of the tree of life, and to track the overall progress of the EBP.

Public databases of the International Nucleotide Sequence Database Collaboration (INSDC: the National Centre for Biotechnology Information’s GenBank, the European Bioinformatics Institute’s European Nucleotide Archive ENA, and the DNA Database of Japan) ^2^ provide resources for the storage of, and access to, submitted reads and assembled genome sequences. While these are the databases-of-record for completed genome projects, it is not the role of the INSDC to coordinate planning of future genome sequencing projects. Until recently, the small scale of eukaryotic genome sequencing efforts meant that there was minimal duplication of effort. As EBP projects scale up to sequencing hundreds of thousands of species, the need for a global platform for registering interest and reporting progress in individual species becomes more pressing.

The Genomes OnLine Database (GOLD) ^3^ registers the intent and status of ongoing and complete genome sequencing projects. GOLD was primarily developed by the US Department of Energy Joint Genomics Institute (JGI) to serve their in-house bacterial, algal, fungal and metagenome sequencing projects. It also periodically imports eukaryotic genome sequencing data from GenBank, but does not aim to be a wide-scope resource to collate genome metadata and live updates from projects from a diversity of sources.

EBP partner projects require access to a diverse suite of genome-relevant metadata to shape and scope sequencing efforts. Estimates of genome size such as those collected by genomesize.com ^4^ and the Kew Plant C Values database ^5^ are valuable for target species selection and determining the amount of raw sequencing data needed to achieve the desired coverage. Karyotypic information and modes of sexual reproduction are also useful to genome curators when piecing together chromosomal assemblies and defining sex chromosomes ^6^. Ideally, data for each attribute would be available for every taxon. However, genome-relevant metadata are sparse and unevenly distributed, with strong taxonomic biases. As a major aim of biodiversity genomics projects is to fill the gaps in the tree of life by sequencing underrepresented taxonomic groups, many of the initial targets of EBP-affiliated projects are among the least likely to have direct measurements of genome size and karyotype available. Coordination of EBP sequencing efforts thus requires a system that automates inference of missing values to provide searchable estimated values for all core attributes across every species.

We have developed Genomes on a Tree (GoaT) to offer public access to plans and progress in genome sequencing at the Tree of Life programme at the Wellcome Sanger Institute, and to offer coordination across biodiversity genomics projects in the wider EBP network. GoaT is built on a flexible, high performance platform called GenomeHubs ^7^ that indexes metadata, infers missing values, and allows rapid querying across indexed attribute values for biological taxa at any taxonomic level. GoaT is a specialised instance of the GenomeHubs platform for genome project planning and status tracking. However, GoaT can also be used as an analytical engine to discover new patterns in genomic features across taxa as the quantity and quality of available metadata increases.

Here we describe the core functioning of GoaT and how we import taxon-specific metadata into GoaT. We show how we use the underlying GenomeHubs platform to make taxonomically informed inferences and support querying across any combination of attributes. We summarise the data available in GoaT at the time of writing and present a set of worked use cases including examples of how GoaT is being used to plan sequencing projects, how other EBP sequencing projects can use GoaT, and how GoaT can be used as a taxonomically-informed search engine. We end with a discussion of how GoaT has rapidly grown to meet the needs of the eukaryotic genome sequencing community, and how we plan to keep extending GoaT to index additional metadata across hundreds of thousands of species.

## Methods

### Core architectural design

GoaT has been implemented as the first instance of our recently updated GenomeHubs codebase ( https://github.com/genomehubs/genomehubs). GenomeHubs provides a flexible data model, search implementation and user interface that has been customised to deliver GoaT through the Configuration-as-Code (CaC) paradigm, where the same code can be extended to different data sources, data types, front ends, and use cases based on human-readable configuration files in the code repository. In the following sections we outline the GoaT-specific customisations and describe the underlying GenomeHubs implementation that GoaT is built upon.

### Data sources and curation

The metadata stored in GoaT are imported from diverse sources. The original data are retrieved in a variety of formats. GoaT currently imports genome and species metadata data dumps from source databases, retrieves information from public APIs, and reads tabular files from manuscripts. To report genome project activity, GoaT uses resources provided by each individual project within the EBP network, largely accessing online spreadsheets that report sequencing planning and progress. Any dataset can be added to GoaT, provided attribute values or descriptors are linked to a unique taxon identifier. These unique taxonomic identifiers are either an NCBI taxon ID ^8^ or a species scientific name plus a higher taxonomic rank to distinguish non-related homonyms.

### Taxonomy

Data import begins with a known taxonomy of eukaryotic species. Because all EBP genomes will be deposited in INSDC databases, GoaT primarily uses the NCBI taxonomy tree ^8^ and NCBI taxon identifiers to align with public sequence databases. Since the NCBI taxonomy only includes taxa with sequence data, it is less comprehensive than some alternative systems such as the Global Biodiversity Information Facility (GBIF) backbone taxonomy ^9^, or the Open Tree of Life Taxonomy (OTT) ^10^. In order to deliver data in the context of a taxonomy of all species, GoaT uses higher taxonomic information to insert additional taxa into the backbone taxonomy at the nearest matching genus, family or other rank. The choice of backbone taxonomy and the method of including additional taxa affects the inference of missing values. The GenomeHubs codebase supports the indexing of values against alternate taxonomies. We intend to index data against additional taxonomies, such as the OTT, in the future.

### Tabular and YAML files

**Figure 1.**
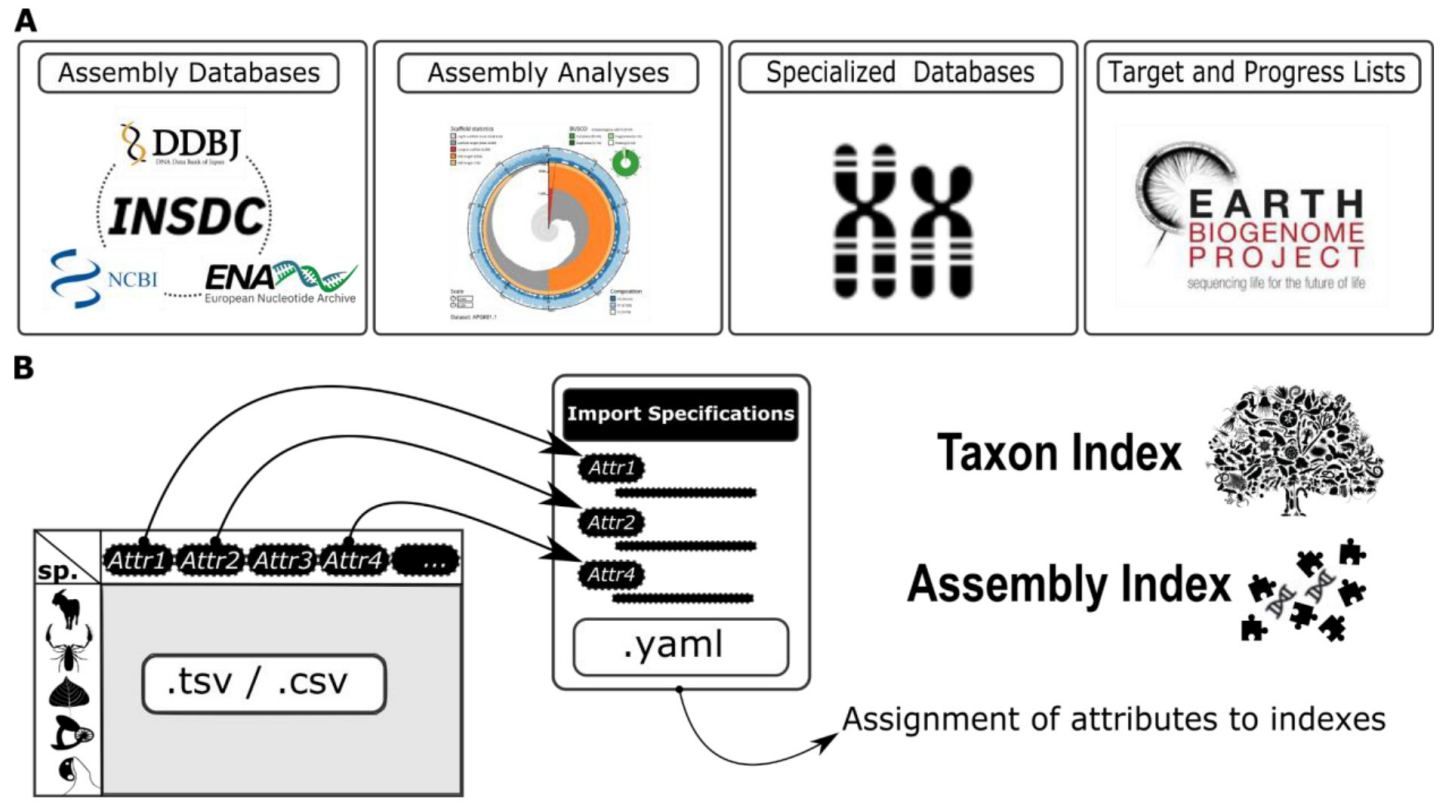
Schematic of the curation process. GoaT data curation can be divided into two phases: ( **A**) Retrieval of taxon and assembly metadata in multiple formats from different sources; ( **B**) Standardisation of metadata to tabular format and preparation of corresponding import specification file. During preparation of TSV and YAML file pairs, curation includes normalisation of values, translation of attribute terms, mapping of columns to existing GoaT attributes, and defining the method for propagation of estimates.

Once the backbone taxonomy has been loaded, GoaT checks out a public GitHub repository (genomehubs/goat-data) consisting of a set of tabular data files and corresponding metadata files that describe how to import the data from each tabular file ( Figure 1). The tabular files may be tab-separated (TSV format), comma-separated (CSV format) or use any other delimiter. For brevity we use TSV below to refer to all tabular file formats. Each TSV file contains one taxon per row, and each column represents a metadata attribute for that taxon, such as its genome size, its cytologically determined chromosome number, or its sequencing status within an EBP initiative. The same pattern is used for genome assembly data with one assembly per row.

**Figure 2.**
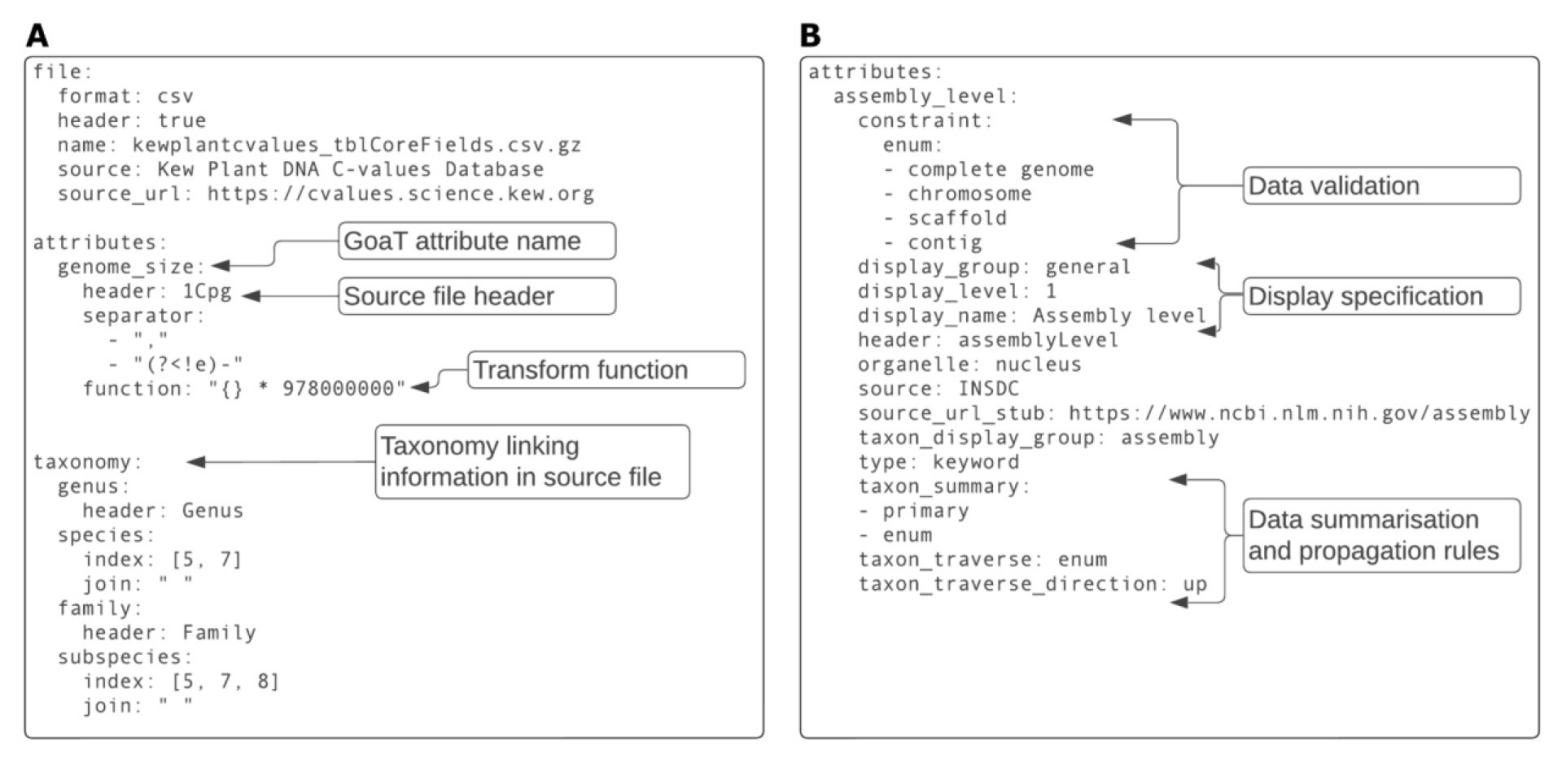
Examples of the Configuration-as-Code (CaC) paradigm. ( **A**) Extracts from a YAML file for describing the Kew Plant C-values Database tabular data source, which specifies the file to be imported, the attributes it fills, what taxa each row refers to, and how the value in the table should be transformed ( Link A). ( **B**) Extracts from a YAML file for describing INSDC assembly attributes in general, how they should be processed and propagated, and how they should be displayed ( Link B).

The TSV-plus-YAML file pairs provide all the required information to define the appropriate data model through the CaC paradigm, offering flexibility to access data processing steps such as renaming attributes, combining columns, validating entries, adding constraints, and translating values to standard vocabularies during data import without writing dedicated code for each attribute. For example, if a particular source provides genome sizes in c-values, GoaT will convert that column from measurements in picograms (pg) to base-pairs (bp) by adding a simple computation function step ( Figure 2). Configuration-as-Code allows these data processing steps to be easily modified, examined and versioned.

For major, stable public data repositories where data are updated regularly, GoaT uses API calls and custom code to generate the import files. For literature sources where import is generally a process-once event, GoaT curators provide custom parsing. For retrieval of data from genome project centres, GoaT curators work to promote shared reporting standards and validation tools. GoaT curators maintain a set of publicly available scripts and workflows in the same GitHub repository that automate the conversion of data from external sources such as APIs, Google Spreadsheets, or other publicly hosted files into tabular files ready for import. A complete list of current data sources and the attributes contributed by each can be reviewed online at goat.genomehubs.org/sources and is summarised in Table 1.

**Table 1.** Numbers of attribute values by source type ( link).

Type of source	Sources	Number of values
Assembly metadata	INSDC; NCBI; BlobToolKit	372,600
Genome- relevant metadata	BUSCOv5; Plant Chromosome Counts Database; Animal Chromosome Counts Database; Coleoptera Karyotype Database; Bird Chromosome Database; Polyneoptera Karyotype Database; Mark Blaxter, Darwin Tree of Life; Bee Chromosome Database; Publications compiled by GoaT data curators and public; Kew Plant DNA C-values Database; Animal Genome Size Database; Amphibian Karyotype Database; de Vos *et al.* 2020; Wioletta Wieloch 2006; Fungal Genome Size Database; DTOL Plant Genome Estimates Kew; Darwin Tree of Life Assembly Team	200,580
Sequencing status	Declared Target List - DTOL UKSI; Declared Status List - B10K; Declared Target and Family Representatives List - DTOL; DTOL Vascular Plants Collections List; DTOL Bryophyte Collections List; DTOL Arthropod Family Representatives List; Declared Status List - CanBP; Declared target list - ASG; Declared Status List - VGP; Declared Target List - VGP; Declared Status List - METAINVERT; Declared Status List - Ag100Pest; Declared Status List - CCGP; Declared Status Lists - EBPN; DToL Protists in Culture List; Declared Status List - Zoonomia; Declared Status List - GAGA; Declared Status Lists - ERGA Pilot; Declared Status Lists - Bioplatforms Australia; Declared Target List - EIN; Declared Status List - GIGA; Declared Status List - CBP; Declared Status Lists - EUROFISH; Declared Status List - SQUALOMIX; Declared Status List - CFGP; Declared Target List - AfricaBP; Declared Status List - ILEBP Pilot; Declared Status List - PGP; Declared Status Lists - ENDEMIXIT; NHM Data Portal - DTOL Samples; Declared Status Lists - DTOL STS	189,925
Legislation	Council Directive 92/43/EEC 1992 Annex IIb Plants; Council Directive 92/43/EEC 1992 Annex IVa Animals; Council Directive 92/43/EEC 1992 Annex IVb Plants; Irish Statute Book Wildlife Act, 1976 Third Schedule (Exceptions); Irish Statute Book Wildlife Act, 1976 Fifth Schedule; Wildlife and Countryside Act 1981 Schedule 5 Animals; Wildlife and Countryside Act 1981 Schedule 1 Birds; The Conservation of Habitats and Species Regulations 2017 Schedule 2; The Conservation of Habitats and Species Regulations 2017 Schedule 5; The Conservation of Offshore Marine Habitats and Species Regulations 2017 Schedule 1; Protection of Badgers Act 1992	1,838
Location	British Ornithologists' Union, The British List; Irish Rare Birds Committee; UK Cetacea; Ireland Cetacea; UK Bats; Ireland Bats	1,256

### Data model

GenomeHubs is built around a denormalised, noSQL data structure implemented in Elasticsearch ^11^, which provides a layer of abstraction over a document-level Lucene ^12^ index. While this involves significant data duplication relative to storage in a traditional normalised SQL database, using an Elasticsearch index as the primary data store allows efficient searches across all data in a GenomeHubs instance such as GoaT and negates the need for a separate index on top of an SQL-based storage layer. Structured information is held in the document store using a single JSON format document per taxon or assembly. Taxon and assembly documents are held in separate indexes to enable efficient querying at either level, and further data are held in additional indexes (described below), so a GenomeHubs Elasticsearch store comprises a collection of searchable indexes.

GenomeHubs data structure is highly suited to GoaT as the data are organised hierarchically on a tree (currently the NCBI taxonomy tree). Each taxon is indexed with a taxon ID, taxon rank and scientific name, in addition to a list of synonyms and a lineage stored as an ordered list of ancestral taxa from the immediate parent through to the root taxon. The synonyms in GoaT are currently taken from the NCBI Taxonomy and from the OTT. Each node in this lineage has taxon ID, taxon rank, scientific name and depth values to facilitate lineage-based searches.

In addition to the taxonomy information, each document in the taxon and assembly indexes has a list of attribute values, such as chromosome number, C-value, or genome assembly span, that are mapped to Elasticsearch data types. Because there are potentially many raw values for each attribute, such as many C-value estimates for a species from different publications, they are stored as lists of raw values. These lists are subsequently summarised following defined rules to generate a single reported value per taxon (see section “genomehubs fill” below). Each raw value is also linked to associated metadata such as the data source. The assembly index has an additional list of identifiers containing assembly aliases and accessions.

Further metadata for each attribute is stored in an attribute index, such as the data type, data constraints, and how the raw values should be summarised and displayed. All these metadata are defined using structured data from the YAML format files generated during data curation or, for selected data sources, through the GenomeHubs command line interface (see below). Finally, details of analyses run on individual or groups of assemblies or taxa can be stored in an analysis index, with associated file metadata in a file index to allow rich analytical results to be displayed alongside assembly and taxon records.

### Data import

**Figure 3.**
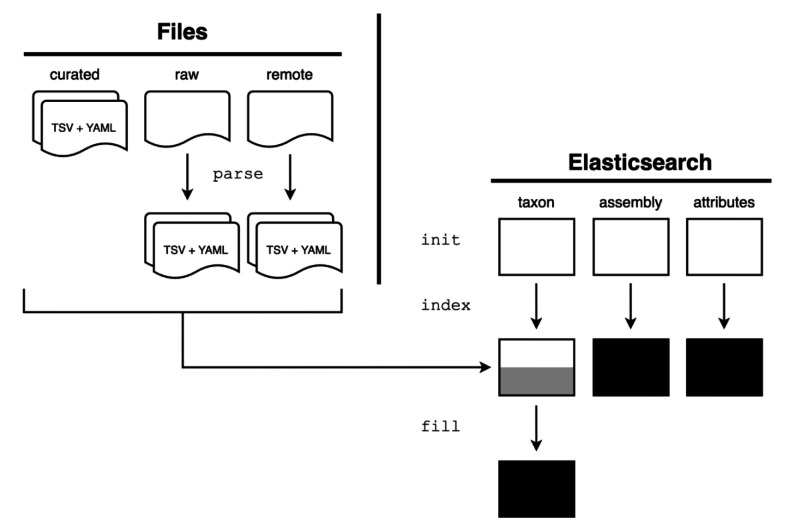
GenomeHubs data import processes. Pairs of data and metadata files (in TSV and YAML formats, respectively) are stored in a separate goat-data GitHub repository. The GenomeHubs *init*, *index*, and *fill* commands use these to create Elasticsearch indexes.

GoaT uses the GenomeHubs Python package, which provides core features for taxon and assembly indexing using the Elasticsearch Python client. The four key GenomeHubs commands are: *init*, *parse*, *index* and *fill* ( Figure 3).

**genomehubs init**

The *init* command initialises the Elasticsearch database with taxonomy, taxon, assembly, attribute, file, and analysis indexes. For each taxonomy it downloads and indexes the latest version and copies the taxonomy structure into the taxon database.

**genomehubs parse**

The *parse* command automates curation processes for selected resources to generate YAML and TSV format files ready for import. Currently supported resources are NCBI Datasets ^13^ to obtain nuclear genome assembly metadata, RefSeq Organelles for organellar genome metadata, NCBI BioSamples for assembled genome sample information such as sample sex and sampling location, and BlobToolKit ^14^ as a source of assembly analyses.

**genomehubs index**

The *index* command can be run on a directory containing YAML and TSV files of either taxon, assembly, or sample attributes. Each YAML file is parsed to set the attribute metadata and the parameters for mapping the data in the associated TSV to the relevant attribute types. Each row in the TSV is parsed in the context of the YAML settings, validated and associated with a taxon (either directly if a valid taxon ID is present, or by looking up taxon names for the species) for indexing. Species name collisions are handled by including higher order taxa in the species lookup and misspellings or matches to synonyms are optionally allowed at any level. Inferred taxon matches are written to a separate file for manual review and any rows that could not be unambiguously matched to a taxon are written to an exceptions file for manual review. For sources that introduce new taxa not present in the backbone taxonomy, the index command can be used to create additional taxon entries anchored at the most closely related existing node so associated metadata can be indexed.

**genomehubs fill**

**Figure 4.**
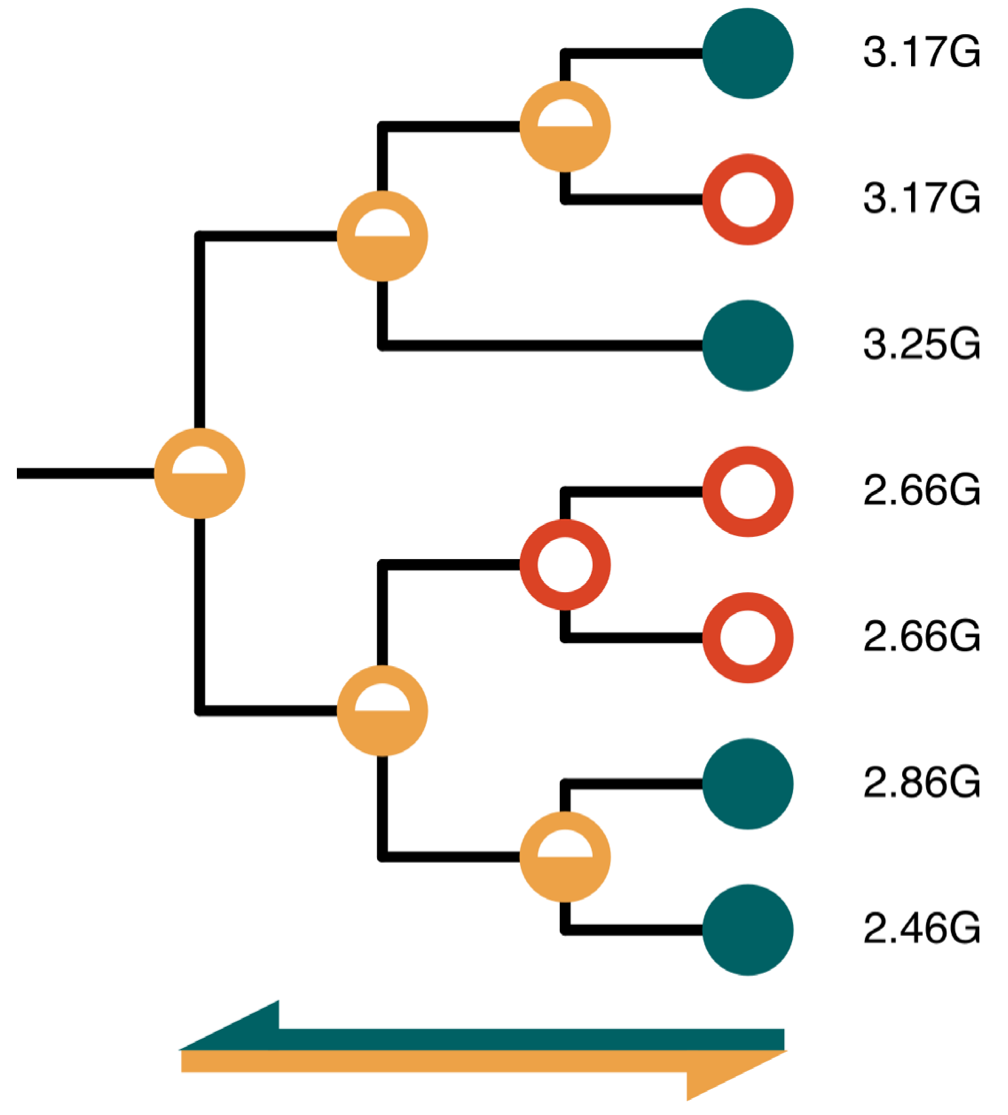
Taxonomically-informed inference of missing values. Directly measured attribute values (green) are used to infer estimated values for parent nodes (orange). These estimated values are then used to fill any unknown descendant node values (red).

The *fill* command is used to determine a single summary value at each taxon level from the raw values in the taxon index and to infer missing values for attributes in the taxon index. The rules for summarisation are defined for each attribute in the YAML and include summary statistics such as maximum, minimum, mean, median, and modal values. In addition, priority is assigned based on the source, or an ordered list of possible values (e.g. favouring an assembly level of “chromosome” over “scaffold”). Rules for inferring missing values are similar and may be applied either only towards the root, towards the tips, or bidirectionally to fill both tip and internal nodes with values based on those of the closest taxa. As the backbone taxonomies do not have branch lengths, the algorithm for filling is very simple, iteratively using available values to infer values for immediate ancestral nodes then filling descendant nodes with the closest available ancestral value ( Figure 4). Traversal can also be limited by rank, for example to prevent inference of plastid assembly span values for Metazoa.

### Data fetching

**Figure 5.**
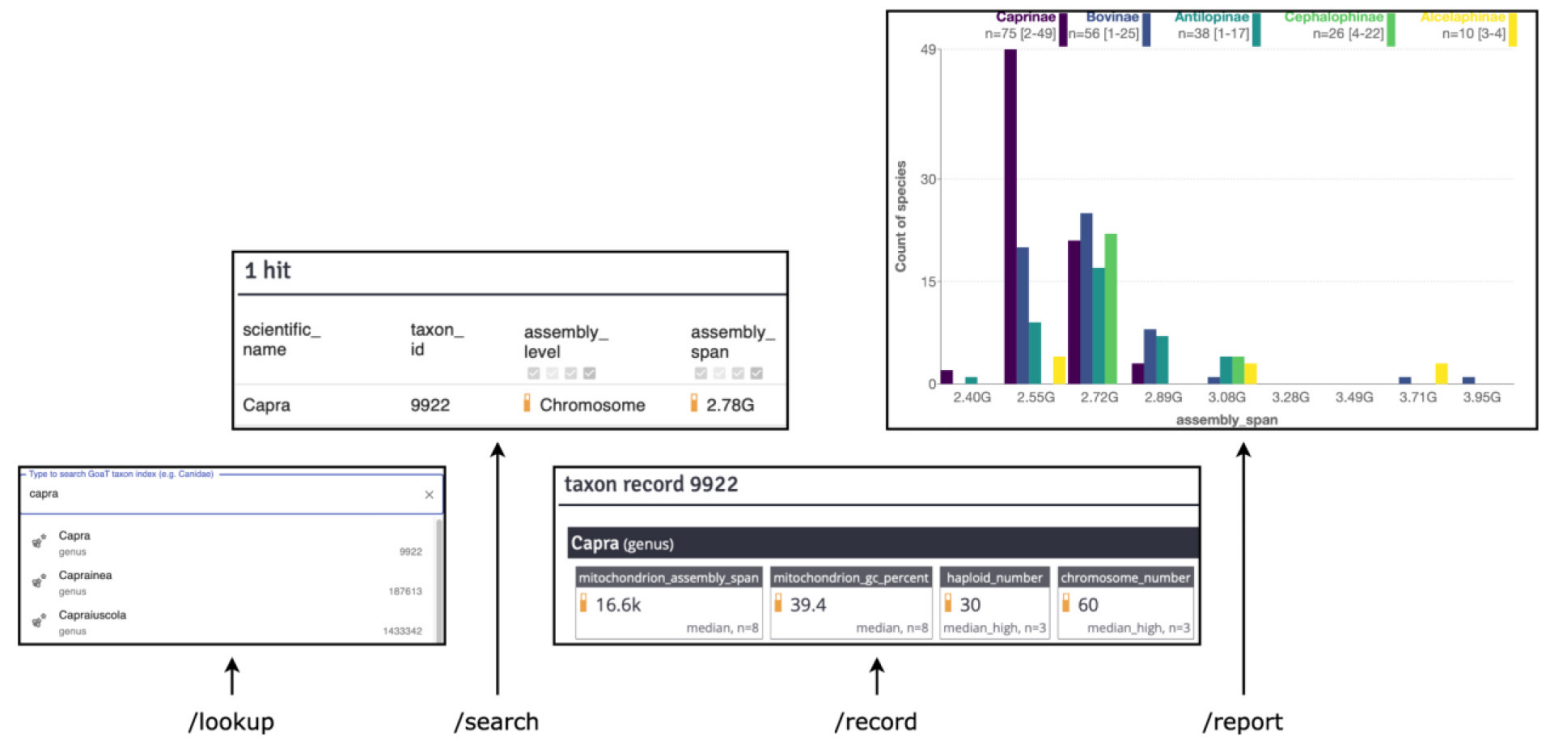
Primary GenomeHubs API/UI endpoints and UI mapping. The autocomplete, search result table, taxon record and report components in the UI map directly to the */lookup*, */search*, */record* and */report* API endpoints, respectively.

The GenomeHubs Application Programming Interface (API) is a Node JS ^15^ application built around the Elasticsearch Javascript client using Express ^16^ and OpenAPI 3 ^17^. The API provides */lookup*, */count*, */search*, */record* and */report* endpoints ( Figure 5) to support both programmatic interaction and the primary functions of a GenomeHubs website. Live API documentation for GoaT is available at https://goat.genomehubs.org/api-docs.

**/lookup**

The */lookup* endpoint provides fuzzy matching of taxon names and assembly identifiers to return unique taxon or assembly IDs for the best matches. For a given query string, results are preferentially returned for exact matches, then prefix matches. If no matches are found then fuzzy matching is used to suggest the most closely matched alternate spellings.

**/count and /search**

The */count* and */search* endpoints both support similar queries to identify matching documents in the Elasticsearch index, but while */search* returns matching results, */count* returns only the number of matches. */search* supports taxon- and attribute-based queries, which can be combined with a logical AND, for example “ tax_tree(Lepidoptera) AND assembly_span > 500000000”. Other boolean operators (OR and NOT) may be used in selected contexts, but are not yet fully supported in user queries. Query values may be joined with commas. Thus “ tax_tree(Lepidoptera, Trichoptera)” will return records matching either Lepidoptera OR Trichoptera. Values may be prefixed with an exclamation mark to exclude matching records, for example “ tax_tree(Lepidoptera, !Nymphalidae)” to return records matching Lepidoptera but NOT Nymphalidae. The != operator may also be used for "is not equal to".

The syntax for taxon-based queries follows that of the European Nucleotide Archive (ENA) portal ^18^ API using functions with a tax_ prefix and including the query value in parentheses. Available functions are tax_eq() and tax_name() for exact matches on taxon IDs and names, respectively; tax_tree() to retrieve all descendant nodes for a taxon ID or name; and additional tax_rank() and tax_depth() functions to restrict results to a given taxonomic rank or depth relative to an ancestral taxon. The tax_lineage() function restricts results to the ancestral nodes of a single taxon up to the root of the tree. Both tax_name() and tax_tree() support name class definitions and wildcards and can be used to retrieve taxa by synonym, for example “ tax_name(synonym:Sophophora melanogaster”, or to retrieve all taxa with a given name class, for example. “ tax_name(tol_id:*)”.

Attribute queries support standard comparison operators (<, <=, =, >=, >, and !=) for numeric values and dates. These operators are also supported for text attributes imported with an ordered list of possible values, otherwise only the = and != operators can be used. Assembly level is an example of such an ordered list where a level of complete genome > chromosome > scaffold > contig. Most taxon attributes include a minimum and maximum from the range of summarised values and these can be queried directly using min() and max() functions, for example “ min(assembly_span)>2000000000 AND max(assembly_date)<2020-11-30”. For list values, the length of the list may also be queried. Thus “ length(long_list)>1” would return taxa that are included on the long lists (the complete list of taxa targeted by a project) of more than one sequencing project.

As values may be directly measured values or estimates based on related taxa, the search endpoint includes further filters to set whether estimated values should be included or not. There are also options to choose which attributes, taxonomic ranks and name classes are included as result fields. Search results are available in JSON, CSV and TSV formats. The TSV format defaults to summary values, but results can optionally be formatted as ‘tidy data’, with one attribute per row, or retrieved as raw values, with only direct measurements for each attribute, one attribute per row.

**/record**

*/record* returns a single document from the Elasticsearch index given a taxon or assembly ID. This allows programmatic access to the full set of attribute values and summary values for each taxon or assembly.

**/report**

*/report* returns structured data based on a query or set of queries and provides summary data for tables, bar charts, histograms, scatter plots and trees of the data in GoaT. Five primary report types are currently available:

- *xPerRank* – summarises the number of results at each rank for a single query
- *arc* – shows the number of results for a query x as a subset of the results of query y
- *histogram* – returns the binned distribution of attribute values for a single query result
- *scatter* – returns paired attribute values for two query results
- *tree* – returns values on a hierarchical tree structure

Reports use Elasticsearch aggregations to allow scaling to millions of values. Aggregations allow summary values to be calculated without the need to retrieve and process each document to extract the information when the query runs. The two exceptions are *scatter* and *tree* reports, which both need to return unprocessed values. For these report types a threshold can be set for the number of results to return. *Scatter* reports for queries that would return more than the threshold number of values use nested histogram aggregations to return a heatmap in place of the raw x/y values. Nested histogram aggregations are also used to allow both histogram and scatter report data to be grouped by an additional category attribute.

The default JSON format for all report types is structured to be efficiently consumed by the web user interface (UI). Standard output formats are also available. For example, *scatter* report data can be exported as TSV and *tree* data can be exported as Newick or PhyloXML.

### Data presentation

**Web UI**

The web User Interface (UI) provides a web frontend to access data using the React ^19^ Javascript framework with a Redux ^20^ store. The UI accesses data via the API and while the UI pulls together data from multiple API endpoints, several of the primary UI components and views are mapped directly to individual API endpoints ( Figure 5). The autocomplete functionality of the main search box is provided by the */lookup* endpoint; search result tables are drawn from the */search* endpoint; record pages are a representation of the */record* endpoint and reports show plots generated from the structured data returned by the */report* endpoint.

The correlation between the UI and API extends to a shared URL query string schema. Interactions with the UI update the browser URL to allow the UI state to be restored from a link. For a given UI view, most relevant API options can be modified directly via the query string in the browser address bar.

The UI provides visual cues to the origins of the values presented using a variant of a traffic light schema with hues selected for accessibility across all types of colour-blindness. A green icon is used to show directly measured values, an orange icon indicates an estimated value that has been inferred from descendant values and a red icon indicates an estimated value that has been inferred from an ancestral value. To further improve accessibility, a thermometer-style icon provides redundant encoding. Further information is provided for each attribute value in the record page to show the summary statistic used, the range of directly measured values included in the summary and a link to the external source for each directly measured attribute value.

GoaT-specific UI customisation is provided through a set of Markdown format files hosted in a public GitHub repository (genomehubs/goat-ui) that allow for site-specific text and images to be included. Through using the Markdown-directive proposal, these files can also access a subset of React components to allow for greater control over styling layout and layout compared to basic Markdown. This mechanism also allows reports to be specified and customised in the Markdown. In GoaT we are using this facility to generate project pages for partner projects such as the Darwin Tree of Life ^21^ and the Earth BioGenomes Project ^1^. Each report can also be accessed and customised via a unique URL, allowing live GoaT reports and plots to be embedded in external websites.

Reports presented in the GoaT UI are intended to provide an efficient way to explore data and relationships between taxa and variables. Users wishing to customise the plots beyond the options provided are encouraged to export the raw or summary data for plotting using dedicated software or plotting libraries.

**GoaT CLI**

The GoaT Command Line Interface (GoaT CLI, executable `goat-cli` version 0.2.5) provides command line access to GoaT data using an asynchronous Rust runtime (tokio ^22^) and HTTP client framework (reqwest ^22, 23^). Like GoaT UI, Goat CLI accesses data via the GoaT API endpoints. Queries may be made against either the taxon or assembly indexes. The / *search* API endpoint is used to return any search that may be made in the GoaT UI, whilst / *report* can be queried to return the underlying data used in the GoaT UI visualisations.

Goat CLI builds request URL strings at execution time, and if more than one taxon is specified, the GoaT CLI makes concurrent requests to the GoaT API. By combining flags and options to the program, the user can specify which variables to return, in addition to any of the taxon or result formatting described in the GoaT API section above. Tabular (TSV) output is returned and printed to the standard output for redirection to files or to be piped as part of a command line workflow. The executable has support on all platforms through crates.io ( https://crates.io/crates/goat-cli) and bioconda ( https://anaconda.org/bioconda/goat), or it can be compiled from source using the Rust toolchain.

**Data releases**

Following the CaC paradigm, we schedule releases to begin shortly after midnight UTC every weekday using GitHub Actions from the genomehubs/goat-data repository. This triggers an automated workflow to run on our local compute nodes that pulls a set of Docker images for the latest GenomeHubs code release, fetches updated taxonomy files and runs a complete import. Each GoaT data release name corresponds to the date on which the backbone taxonomy and other data files are retrieved.

We plan to archive releases once every quarter, beginning with the 2022.11.16 release described here to ensure long-term access to GoaT versions used to support published research. Archived releases will remain available at URLs of the form https://goat.genomehubs.org/<archive-version>/, for example https://goat.genomehubs.org/2022.11.16/.

## Results

We have developed GoaT as a versatile and stable platform for genome project coordination and broader genomic research. Our data curation processes and use of automation allow us to keep GoaT up to date with updates every weekday to reflect the latest information available in INSDC and other sources. The description provided here is a snapshot of the data available in GoaT archival release 2022.11.16, built using GenomeHubs version 2.5.39. Where we provide links to interactive versions of Figures and Tables below, the latest version can be accessed by clicking on the archive name in the site header.

### Numbers of taxa and assemblies in GoaT

**Figure 6.**
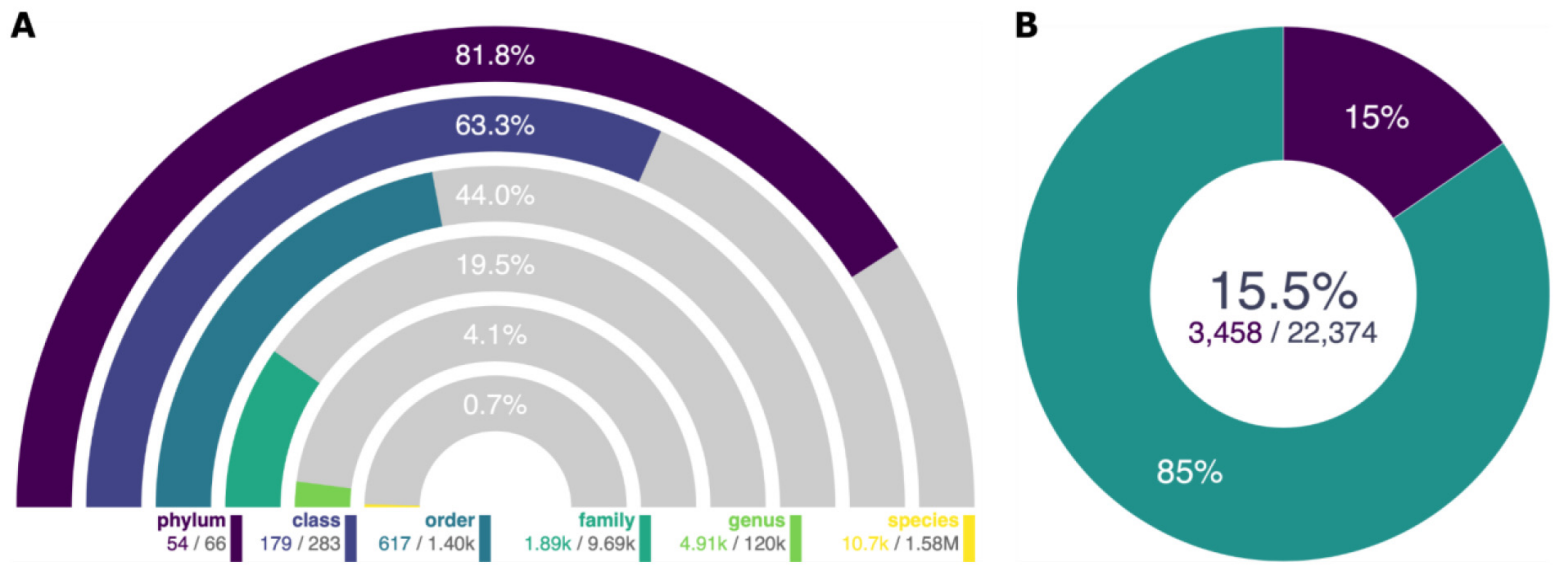
Numbers of taxa and assemblies in GoaT. ( **A**) Number of taxa with publicly available assemblies out of all taxa at ranks from phylum to species ( Link A). ( **B**) Number of chromosomal or complete genome assemblies out of all assemblies ( link B).

As of November 16 ^th^ 2022, GoaT has information for 1,826,192 taxa across all ranks, including 1,583,871 species of which 10,678 (0.7%) have publicly available genome assemblies ( Figure 6A). Some taxa have multiple assemblies so GoaT has a total of 22,374 assemblies, of which 3,458 (15.5%) are labelled chromosomal or complete ( Figure 6B).

### Summary of GoaT attributes and data sources

Values in GoaT are derived from 68 sources which provide a total of 766,199 values ( Table 1) across 77 taxon attributes with 14,562,377 estimated values at the species level inferred from ancestral taxa ( Table 2). All 33 assembly attributes ( Table 3) are derived directly from INSDC records.

**Table 2.** Counts of directly recorded and estimated values at the species level for each taxon attribute in GoaT.

Display group	Attributes	Count of direct values *	Count of estimated values
assembly	assembly_level; assembly_span; bioproject; biosample; contig_n50; assembly_date; scaffold_n50; gene_count; ebp_metric_date; btk_nohit; btk_target; busco_completeness; busco_lineage; busco_string; gc_percent; n_percent; odb10_lineage	122,242	3,150,416
genome_size	c_value; c_value_method; c_value_cell_type; genome_size; genome_size_kmer; genome_size_draft	53,880	3,135,351
karyotype	chromosome_number; haploid_number; sex_determination; ploidy	94,403	4,659,266
mitochondrion_assembly	mitochondrion_assembly_span; mitochondrion_gc_percent	28,194	3,119,060
plastid_assembly	plastid_assembly_span; plastid_gc_percent	20,460	488,218
regional_lists	country_list	689	0
sample	sample_sex; sample_location	2,949	0
sequencing_status	sample_collected; sample_collected_by; sample_acquired; in_progress; insdc_open; published; sequencing_status; sequencing_status_ag100pest; sequencing_status_asg; sequencing_status_b10k; sequencing_status_canbp; sequencing_status_cbp; sequencing_status_ccgp; sequencing_status_cfgp; sequencing_status_dtol; sequencing_status_ebpn; sequencing_status_ endemixit; sequencing_status_erga; sequencing_status_eurofish; sequencing_ status_gaga; sequencing_status_giga; sequencing_status_ilebp; sequencing_ status_metainvert; sequencing_status_pgp; sequencing_status_squalomix; sequencing_status_vgp; sequencing_status_zoonomia; sequencing_status_ omg; sequencing_status_arg; sequencing_status_agi; sequencing_status_tsi; sequencing_status_gap; sequencing_status_gbr; sequencing_status_ebp	75,790	10,066
target_lists	long_list; other_priority; family_representative	87607	0
uk_legislation	echabs92; habreg_2017; marhabreg-2017; waca_1981; isb_wildlife_act_1976; protection_of_badgers_act_1992	1733	0

*The count of directly recorded values includes species for which the value is associated with a descendant subspecies.

**Table 3.** Assembly attributes in GoaT by display group.

Index	Display group	Attributes
assembly	annotation	gene_count, noncoding_gene_count, protein_count, pseudogene_count
assembly	btk	btk_nohit, btk_target
assembly	busco	busco_completeness, busco_lineage, busco_string
assembly	general	assembly_level, assembly_type, bioproject, biosample, ebp_metric_date, isolate, last_ updated, organelle, refseq_category, sample_sex, submitter, sample_location
assembly	metrics	assembly_span, chromosome_count, contig_count, contig_l50, contig_n50, scaffold_count, scaffold_l50, scaffold_n50, sequence_count, ungapped_span, gc_percent, n_percent

### Summary of listed genome projects

GoaT currently has target lists for 25 sequencing projects in the EBP network (summarised in Table 4). To facilitate creation of summary reports on GoaT, our recommendation for new projects is to create project reference numbers on INSDC (BioProject ID or equivalent), linked to the EBP umbrella bioProject ID PRJNA533106. 

**Table 4.** List of Earth BioGenome Project (EBP) Affiliate projects with data on GoaT with species and assembly counts.

Project name	Acronym	BioProject ID	Scope	Count of target species listed on GoaT			
				Targets	With assemblies	With EBP standard assemblies *	Assemblies under the EBP umbrella BioProject
African BioGenome Project (AfricaBP)	AFRICABP	PRJNA811786	Eukaryotes	12	4	4	0
Ag100Pest Initiative	AG100PEST	PRJNA555319	Arthropods	160	54	19	11
Australian Amphibian and Reptile Genomics Initiative Collaboration (AusARG)	ARG	-	Amphibians and reptiles	20	3	0	0
Aquatic Symbiosis Genomics Project	ASG	PRJEB43743	Eukaryotes and symbionts	348	32	4	6
Bird 10,000 Genomes Project	B10K	PRJNA545868	Aves	8,207	585	62	216
Canada BioGenome Project (CBP)	CANBP	PRJEB49670	Eukaryotes	612	54	9	0
Catalan BioGenome Project	CBP	PRJEB49670	Eukaryotes	32	7	2	1
California Conservation Genomics Project (CCGP)	CCGP	PRJNA720569	Eukaryotes	146	64	4	40
Cartilaginous Fish Genome Project	CFGP	-	Chondrichthyans	19	0	0	0
Darwin Tree of Life (DToL)	DTOL	PRJEB40665	Eukaryotes	72,029	2,630	649	435
Earth BioGenome Project Norway (EBP-Nor)	EBPN		Eukaryotes	136	34	3	0
Italian Endemics	ENDEMIXIT	PRJNA712951	Vertebrates + Lepidoptera	5	1	0	0
European Reference Genome Atlas (Pilot project)	ERGA	PRJEB47820	Eukaryotes	92	7	2	1
The Euro-Fish Project at the MPI CBG	EUROFISH	PRJNA393850	Freshwater fish	24	20	19	0
Global Ant Genomics Alliance	GAGA	-	Ants	130	8	2	0
Genomics for Australian Plants	GAP	-	Plants	10	4	2	0
Global Invertebrate Genome Alliance	GIGA	PRJNA649812	Invertebrates	44	44	12	1
Illinois EBP Pilot	ILEBP	PRJNA844590	Insects	9	0	0	0
Soil Invertebrate Genome Initiative	METAINVERT	PRJNA758215	Invertebrates	203	24	2	0
Oz Mammals Genomics	OMG	-	Mammals	15	3	0	0
Polar Genomes Project	PGP	-	Eukaryotes	8	2	0	0
Genome Sequencing and Assembly of Chondrichthyans	SQUALOMIX	PRJNA707598	Chondrichthyans	20	4	0	4
Threatened Species Initiative	TSI	-	Eukaryotes	29	2	0	0
Vertebrate Genomes Project	VGP	PRJNA489243	Vertebrates + outgroups	392 **	268	166	157
200 Mammals Project	ZOONOMIA	PRJNA312960	Mammals	125	125	9	124

* EBP standards are contig N50 > 1Mb and scaffold N50 > 10Mb ^24^. ** The VGP has ~70,000 target species but we are currently only tracking the ~400 species at https://github.com/vgl-hub/genome-portal. Status list sources are constantly being updated and revised.

## GoaT use cases

Sequencing genomes at the scale of the EBP requires careful coordination between affiliated projects to prevent duplication of effort and to ensure resources are used efficiently. Metadata stored in GoaT can be used throughout a sequencing project to help meet these requirements. Here we describe a set of use cases from planning to completion that can be applied to any sequencing initiative in the EBP Network and more broadly to any genome sequencing project. The following examples are illustrated with screenshots and images that demonstrate the utility of GoaT. We primarily use the Darwin Tree of Life (DToL) ^21^ as an example project, but the equivalent information can be retrieved for any EBP-affiliated project on GoaT using the relevant acronym, listed on https://goat.genomehubs.org/projects and in Table 4, in place of "DTOL" in the examples below.

### Project planning

Regardless of scope, the initial step for most sequencing projects is to generate a list of target taxa. As Table 4 shows, projects may delimit their targets to a geographic region (e.g. DToL, EBPN, CBP, ERGA, etc.) or to a taxonomic group (e.g. B10K, Bat1K, CFGP, VGP, etc.), but these target lists will often overlap. For projects that submit declarations of sequencing intent and priority, GoaT can provide a platform to make these public to enable searches across lists of multiple projects. Used in this way, GoaT can help to identify overlapping taxa and provide a catalyst for liaison between working groups.

GoaT stores three categories of target lists to help distinguish taxa that are likely to be sequenced in the long- versus short-term: *long_list* includes all targets of a project, whereas *family_representativ*e and *other_priority* are subsets of species in the *long_list* that will be prioritised. The *family_representative* list reflects the EBP’s phase 1 goal of sequencing one species for each of the ~9,000 taxonomic families of Eukaryota. A sequencing project will want to refine its target list to remove any species for which an assembly meeting the project's criteria is already available (Case 1), coordinate efforts in instances of overlap with different sequencing initiatives (Cases 2 and 3), identify gaps in taxonomic coverage to target the most distinct taxa (Case 4) and finally to remove taxa from priority lists if the genome metadata suggest they may be outliers (Case 5).

**Case 1 - Which species on a target list have already been sequenced to the desired standards?**

**Figure 7.**
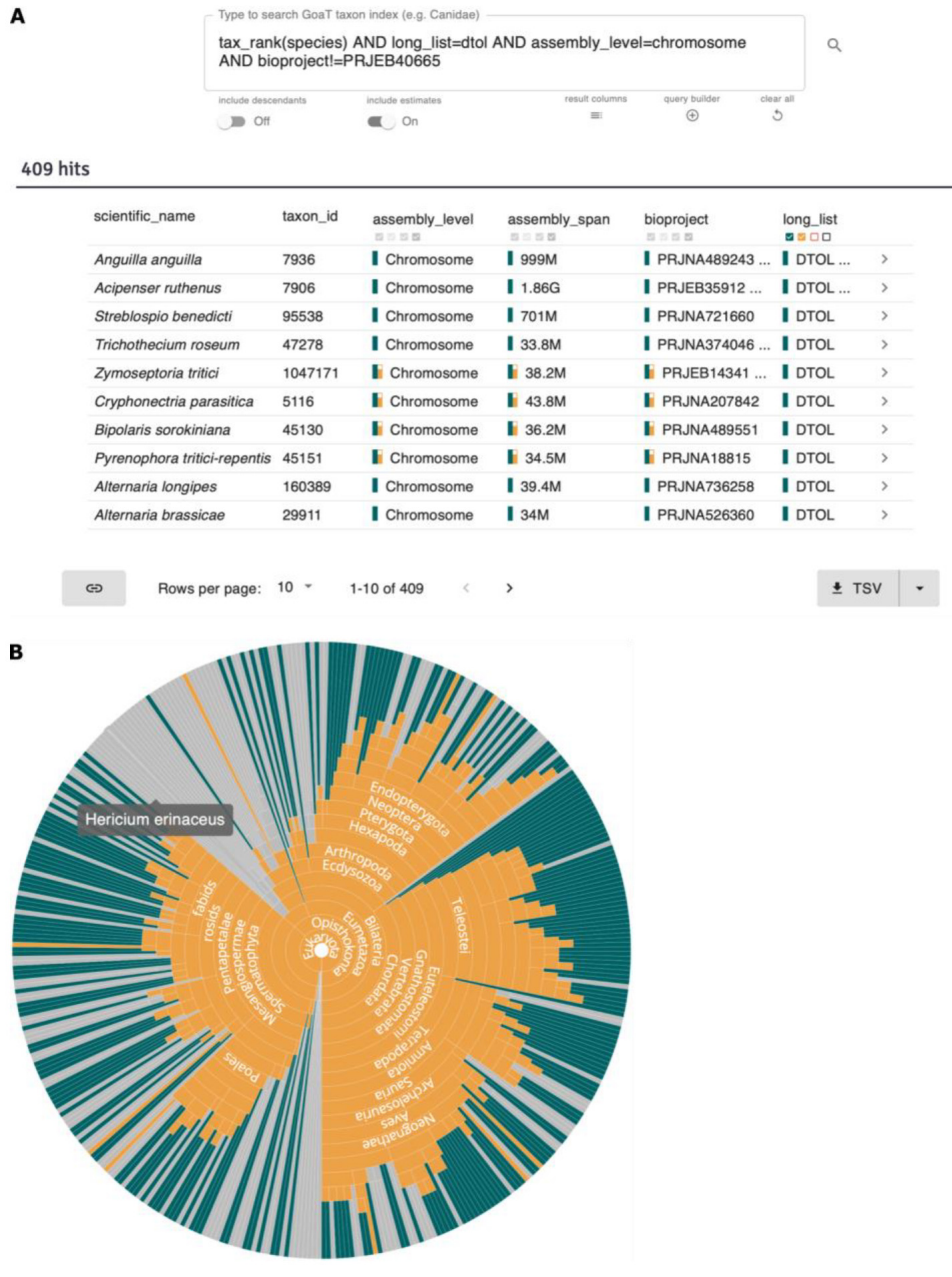
Summary of available chromosomal assemblies for species on the DToL long_list. ( **A**) GoaT UI search result table for the query “ tax_rank(species) AND long_list=DTOL AND assembly_level=chromosome AND bioproject!=PRJEB40665” ( link A). ( **B**) Tree report for the same search, highlighting assemblies that meet the EBP threshold of “ contig_n50>1000000 AND scaffold_n50>10000000” ( link B). Green highlights indicate directly measured values while orange highlights show information derived from a descendant taxon. Tree reports are interactive and taxa can be displayed on tooltips, expanded into subtrees by long-pressing, or redirected by short-clicking to their respective records page.

The DToL project aims to sequence each of the ~70,000 eukaryotic species in Britain and Ireland ^21^. This target list is represented in GoaT using the attribute-value pair “ long_list = DTOL” and currently contains 72,029 species. Some of these target species have already been the subject of genome sequencing efforts and high-quality chromosome-level genome assemblies are publicly available for several hundred species ( Figure 7A).

This list of available assemblies can be reviewed during project planning and may be used to de-prioritise and/or exclude taxa from the project. Further metadata (also stored in GoaT) such as the origin of the sequenced sample and assembly statistics may be used to inform these decisions. Reports are available to combine information into summary views to aid decision making, such as the tree in Figure 7B, which shows the taxonomic distribution of available chromosomal assemblies, highlighting those that meet the EBP standards (contig N50 > 1Mb and scaffold N50 > 10Mb ^24^).

**Case 2 - What species in the DToL long list are also targeted by other projects? Are any shared targets also declared as a priority by another project?**

**Figure 8.**
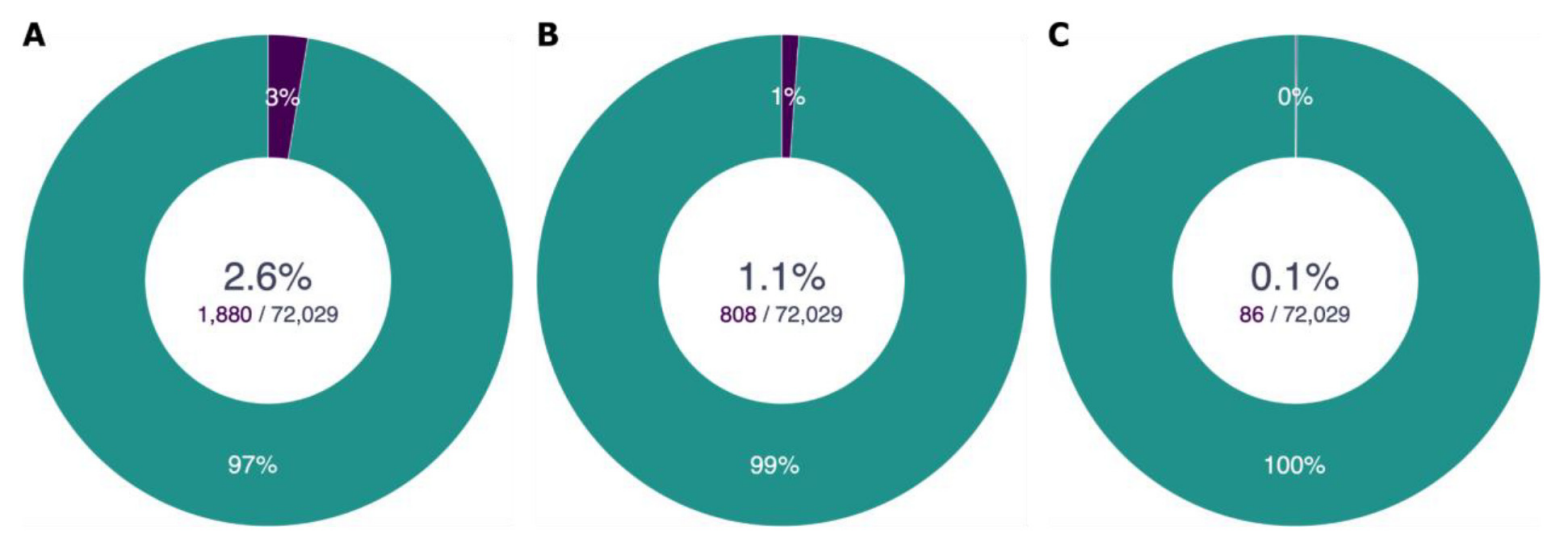
Numbers of DToL target species that are also included on the target list of another EBP-affiliate project. ( **A**) Total number of DToL target species included on any other target list ( link A). ( **B**) Number of DToL target species included on a priority list of another project ( link B). ( **C**) Number DToL target species listed as a family representative by another project, but not by DToL ( link C).

As GoaT stores information about sequencing intent for multiple sequencing initiatives, decisions on prioritisation can also take into account the overlap between different projects. The *long_list* attribute is not restricted to a single value per taxon, but can store a list of values, one for each project that targets that taxon. Therefore it is possible to query the length of the list of values in the *long_list* to identify taxa that overlap between projects using “ length(long_list) > 1” to return any taxon for which there is more than one project represented in the *long_list* attribute. The search can be refined to generate a report of how many DToL target species are shared with other projects, how many are on a priority list for another project and how many are listed as *family_representatives* for other projects ( Figure 8).

**Case 3 - Sequencing status and coordination between sampling working groups and sequencing centres**

**Figure 9.**
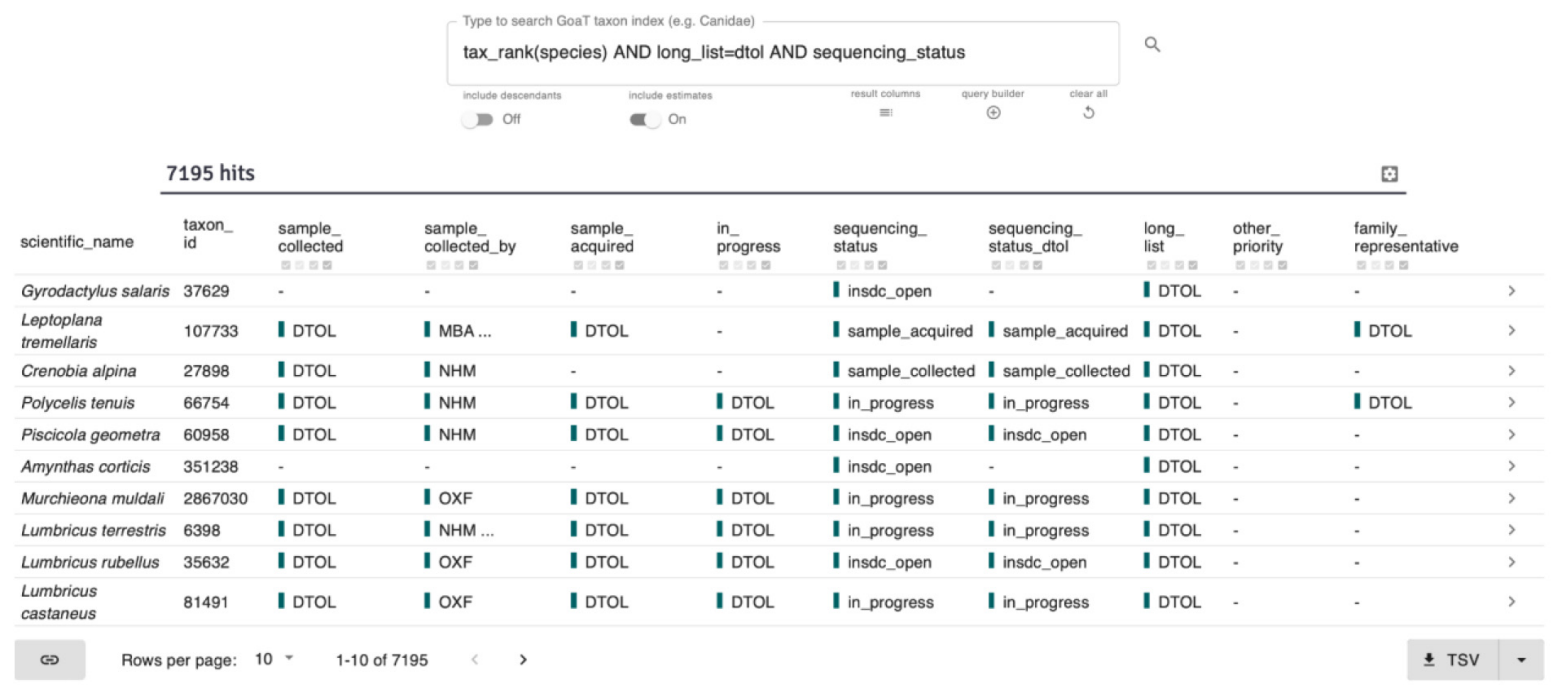
GoaT search result table for the query “ tax_rank(species) AND long_list=dtol AND sequencing_status” showing sequencing status columns for species on the DToL target list ( link).

Following initial prioritisation, the focus of planning changes and requires monitoring of progress within a project and the wider suite of EBP-affiliate projects. GoaT tracks changes in sampling and sequencing status to provide information to help sampling working groups and sequencing centres coordinate, distribute or halt activity on target taxa. Similar to the target list attributes in Cases 1 and 2, sequencing status attributes allow users to track which projects have reached collection and sequencing milestones for each taxon. The search results in Figure 9 show the 7,195 taxa on the DToL *long_list* that have reached a *sequencing_status* milestone of at least having a *sample_collected* by one or more projects. Each status is represented by an attribute with a list of project names as values alongside a single *sequencing_status* attribute that shows the most advanced status across all projects. A separate summary attribute, *sequencing_status_dtol*, contains project-specific status within the DToL pipeline.

**Case 4 - Identifying gaps in sequencing across the Tree of Life**

**Figure 10.**
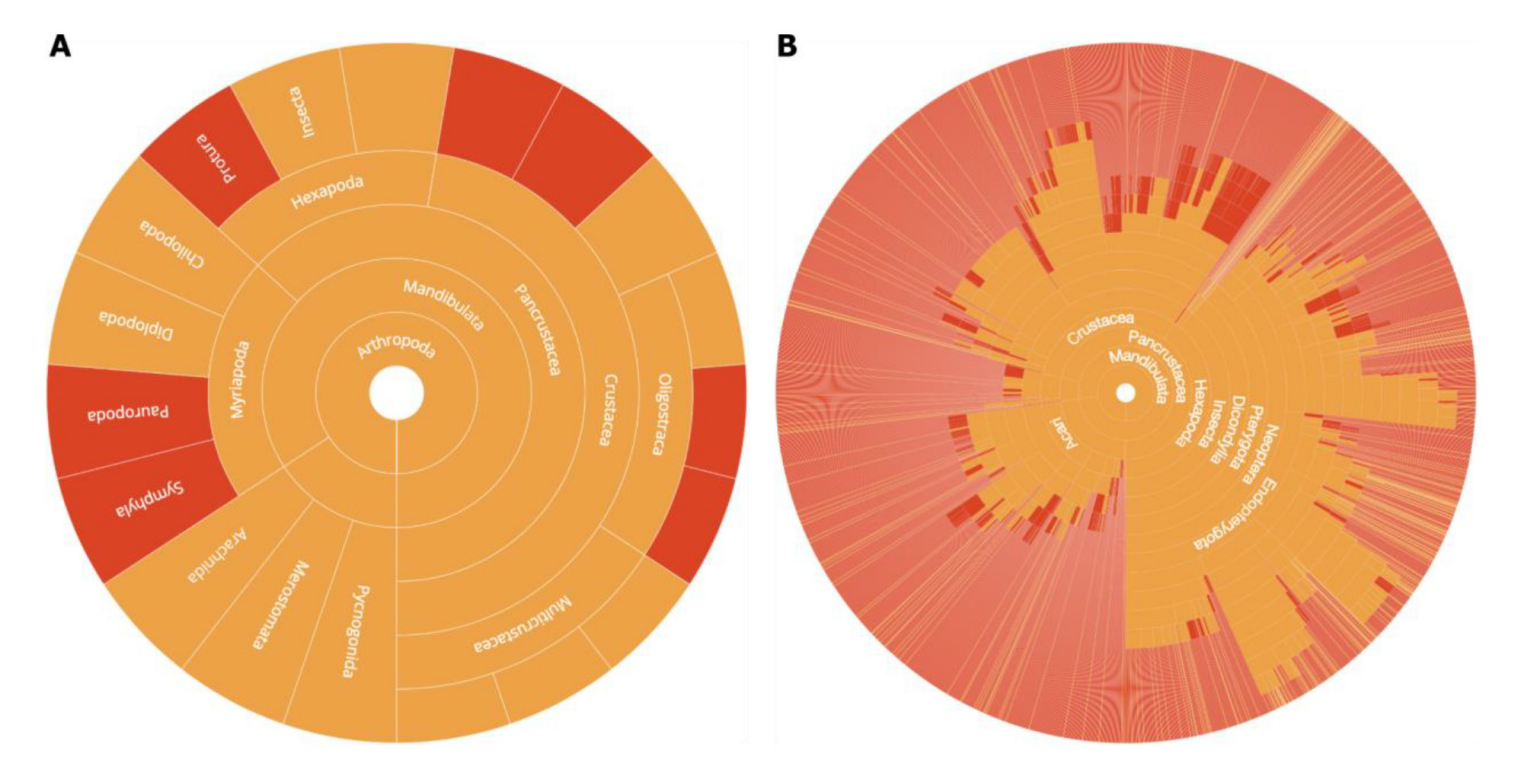
Tree reports showing the distribution of sequencing effort in Arthropoda. Activity summarised ( **A**) by class ( link A) and ( **B**)by family ( link B). Taxa with publicly available genome assemblies for any descendant taxon have an orange highlight and those without have a red highlight. The interactive versions of these plots show tooltips on mouseover to display taxon names for arcs that are too small to accommodate a taxon name in the default display.

In order to make effective use of limited resources, projects may choose to focus their efforts on underrepresented taxa. GoaT facilitates gap analysis to identify these underrepresented taxa and can therefore be an important tool even for early stages of the preparation of grant proposals and target list generation. As more initiatives emerge, similar gap analysis will be increasingly relevant to achieve comprehensive coverage at different taxonomic levels across the wider EBP. Within the Arthropoda, for example, GoaT can be used to visualise underrepresented classes and families in the set of available genome assemblies ( Figure 10).

**Case 5 - Excluding taxa with outlier genome metadata from priority lists**

**Figure 11.**
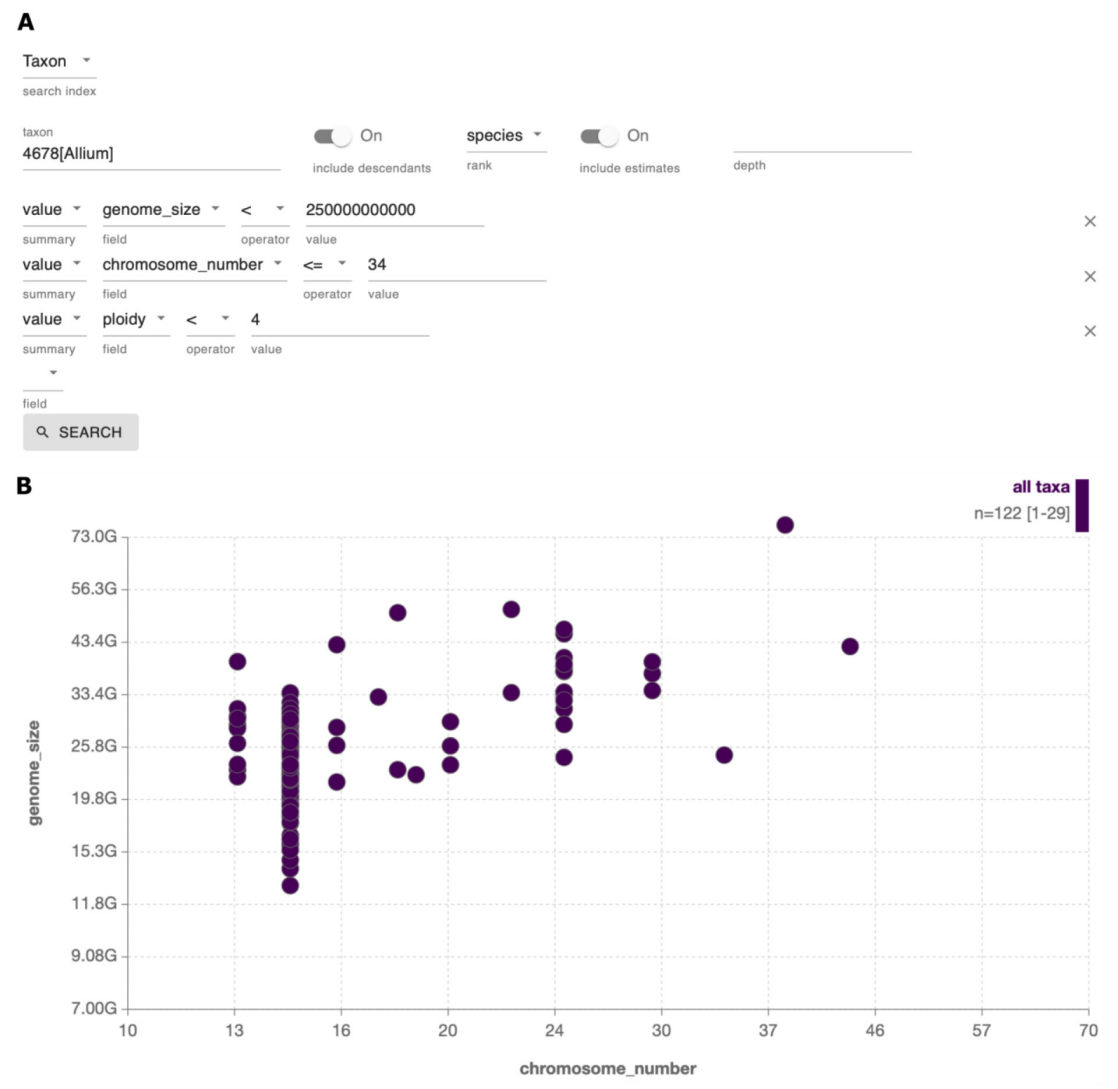
Examples of strategies to remove outliers from target lists. ( **A**) Using the query builder to refine a query to exclude species with large chromosome size and chromosome number above a threshold of 34, and with ploidy greater than 4n ( link A). ( **B**) Exploratory search of *Allium* species with ploidy varying from 2n to 8n ( link B). Taxa with the lowest values for chromosome number and genome size could be selected as genus representatives.

Exclusion of taxa with known outlier values or likely estimates for attributes such as genome size, chromosome number, supernumerary chromosomes and sex determination system can help to maximise the chances of producing high quality reference assemblies. This is particularly true of projects focused on genomics of understudied taxa. The directly measured and estimated values in GoaT can be used to refine priority lists of target species, such as family or genus representatives, either by applying thresholds to queries or performing exploratory searches of pre-selected taxa ( Figure 11).

### Project execution

During the execution of a sequencing project, GoaT metadata can inform all steps from estimation of sequencing effort required for a taxon (Case 6) to interpretation of Hi-C data for curating genomes (Case 7) and quality assessment of the final genome assembly metrics (Case 8).

**Case 6 - Estimation of sequencing effort**

Direct values and estimates of genome size provided by GoaT are routinely used in the DToL project to predict the sequencing effort required for a given taxon. DToL has implemented standard operating procedures for long read (PacBio HiFi), and long range (Hi-C) sequencing coverage required for assembly, and uses the expected genome size value from GoaT to determine the sequencing effort needed to reach the target sequencing depth for each sequencing platform.

**Figure 12.**
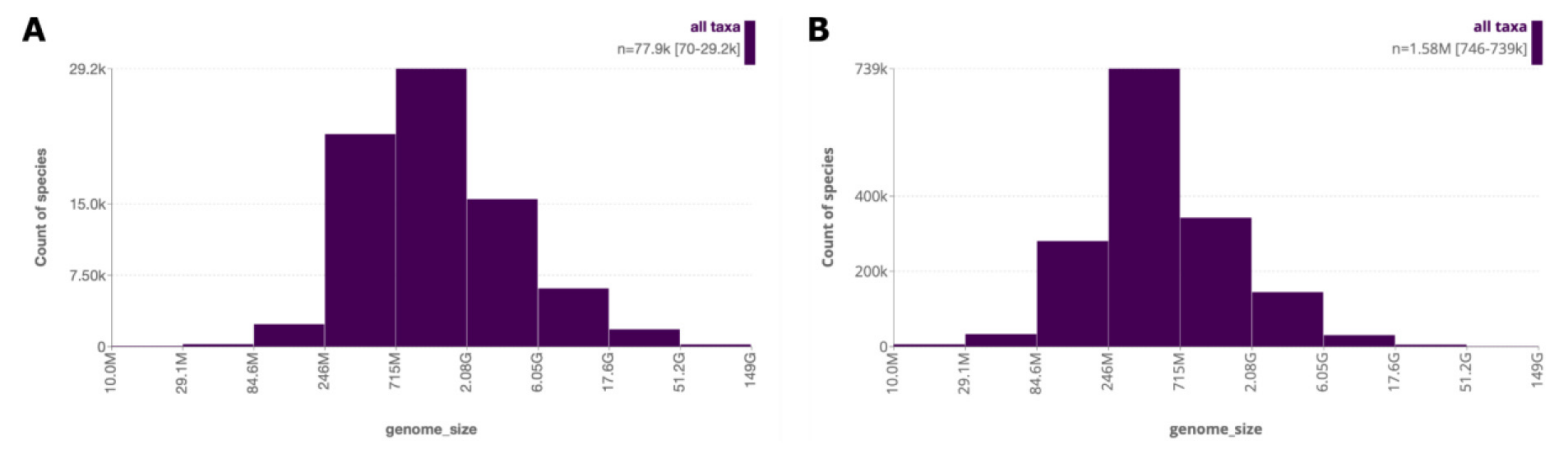
Distribution of genome size values across species in GoaT. ( **A**) showing directly measured values only ( link A), and ( **B**) including estimated values ( link B).

By including estimated values inferred by GoaT, we greatly increase the number of *genome_size* values from 36,000 species to all 1.6 million species in GoaT ( Figure 12). Genome sizes can be retrieved using a list of species as input. Additionally, because estimated values are filled up to the root of the tree, searches can be performed at the level of genus or any higher taxonomic rank to retrieve estimates for taxa not yet present in the backbone taxonomies.

**Case 7 - Interpretation of Hi-C data**

**Figure 13.**
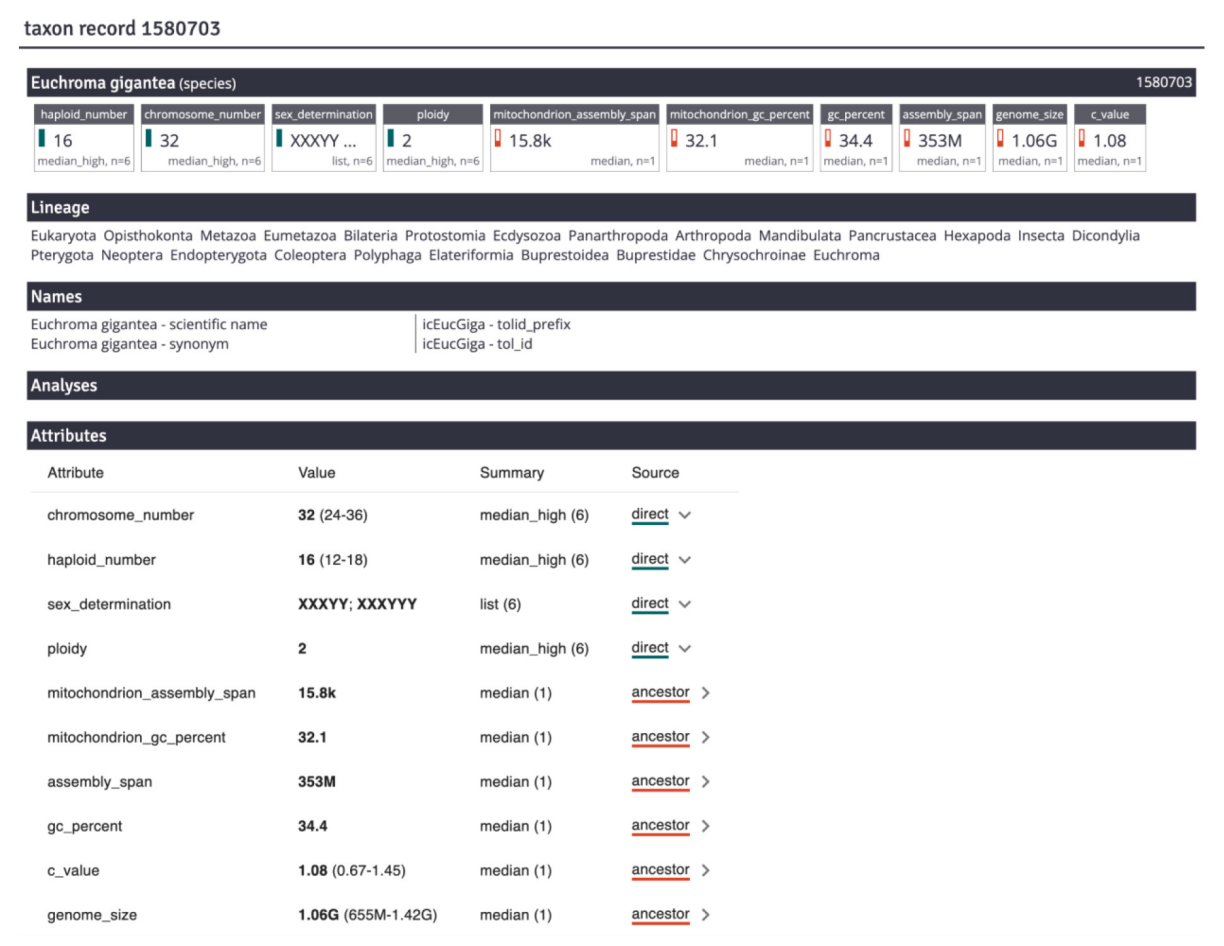
A taxon record page for *Euchroma gigantea*, NCBI Taxon ID 1580703 ( link). This beetle has an unusual sex chromosome system (XXXYY; XXXYYY) and knowledge of this feature aids in resolution of assembly issues.

The DToL production team uses manual curation to create chromosomal assemblies using Hi-C maps and information about expected chromosome number, ploidy, and chromosomal sex determination mechanisms. GoaT provides a single portal for such information on the taxon record pages ( Figure 13). Each taxon record page includes a full list of directly measured and estimated attribute values for the taxon. Details of the source for each attribute value are provided together with a link to allow direct access to the source data used in GoaT.

**Case 8 - Quality assessment of genome assembly and annotation**

**Figure 14.**
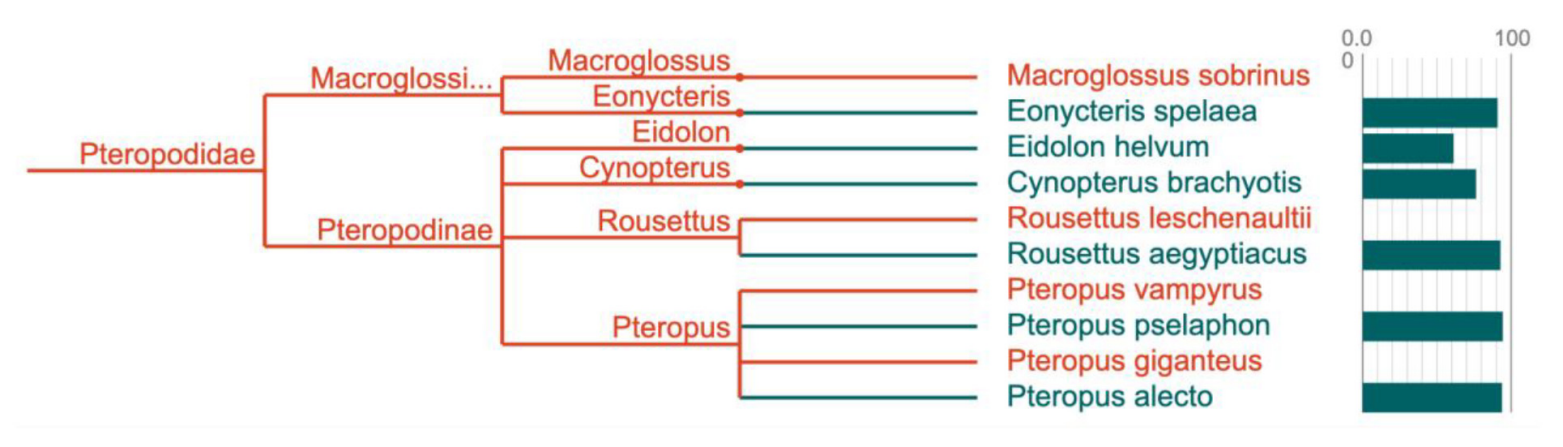
Tree report highlighting the distribution of BUSCO completeness scores among species in the bat family Pteropodidae with publicly available genome assemblies ( link).

Quality control is important throughout the sequencing and assembly pipeline prior to public release. GoaT can be used to compare assembly metrics and scores with those of closely related taxa to provide an estimate of genome quality. For example, biological completeness, estimated using the Benchmarking Universal Single-Copy Orthologs (BUSCO) ^25^ system, varies considerably between lineages for assemblies of comparable quality. GoaT allows these patterns to be visualised on trees of related taxa ( Figure 14) to aid in assessing whether a given score is close to the expected value.

### Project reporting

All GoaT reports are reproducible from the URL, which can be embedded in external web pages or placed alongside text and other reports as part of GoaT UI, using an extended Markdown syntax. This allows GoaT to serve as a reporting platform for EBP-affiliated projects (Case 9) and to be used to generate ad-hoc reports on any of the metadata stored in GoaT, including at the assembly level (Case 10).

**Case 9 - Exploring EBP-affiliated project pages on GoaT**

**Figure 15.**
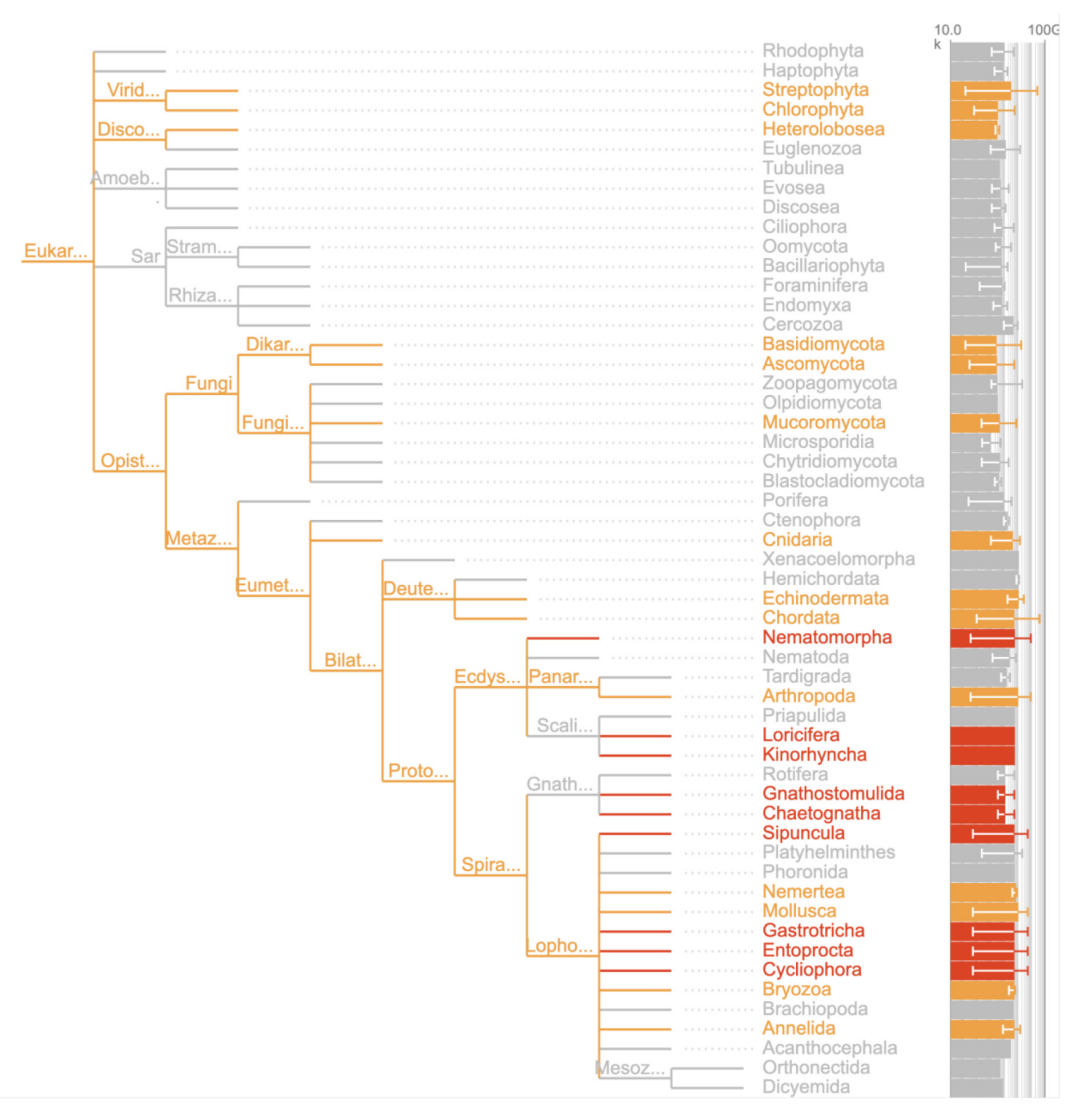
Tree report of phyla on the DToL long list showing representative assembly span of each phylum ( link). Orange highlights show phyla with at least one assembly released under the DToL BioProject (PRJEB40665). Red highlights show phyla with no publicly available assemblies. Taxa in grey have at least one publicly available assembly but none under the DToL bioProject.

GoaT UI hosts dedicated pages for 25 EBP-affiliated projects (see, for example the DToL project page at https://goat.genomehubs.org/projects/DTOL). Each project page contains information about the project including its GoaT search term and BioProject ID alongside reports of planning, progress and completion of genome assemblies. The project page reports follow a common structure beginning with a tree of declared target taxa ( *long_list*) based on the NCBI backbone taxonomy. In most instances, this tree also displays a progress metric or attribute and highlights taxa in progress or those with completed assemblies alongside corresponding assembly span ( Figure 15). Trees are interactive: long-pressing on specific nodes results in expansion of the tree, and updating of the associated result table at the top of the page. Tapping on specific nodes opens the associated taxon record page, with a summary of all attributes for that taxon.

**Case 10 - Generating progress and assembly quality reports for sequencing projects on GoaT**

**Figure 16.**
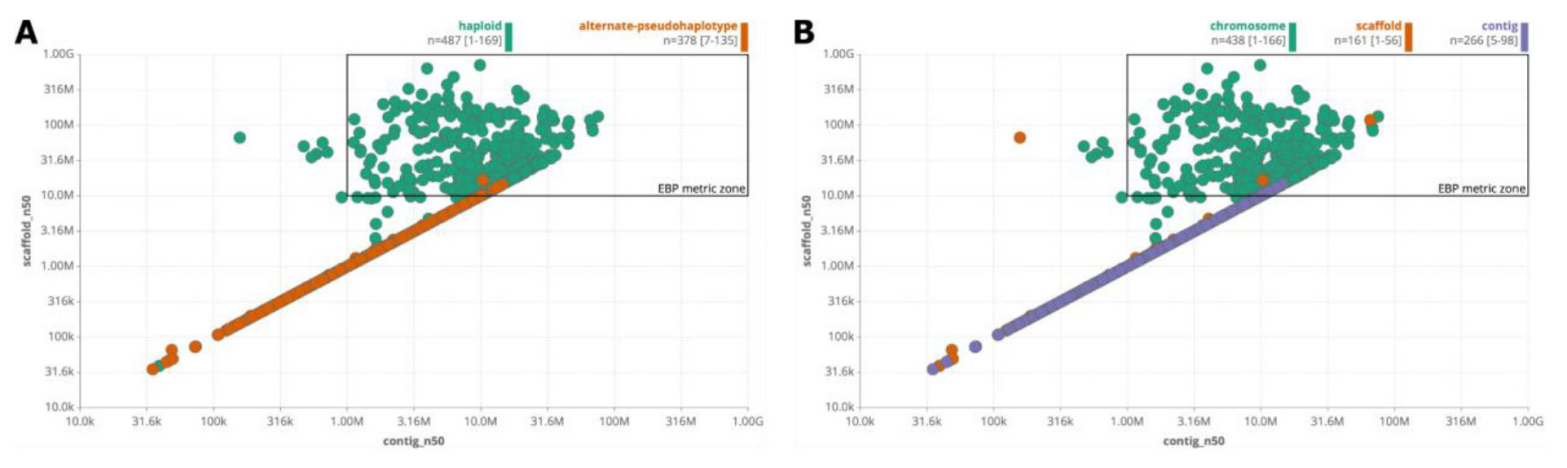
Scatter report showing contiguity assessment of DToL genomes released under BioProject PRJEB40665. The EBP metric zone highlights assemblies with a contig N50 > 1Mb and a scaffold N50 > 10Mb, ( **A**) highlighting assembly type with primary haploid assemblies are shown in green and alternate haplotypes in orange ( link A). ( **B**) highlighting assembly level with chromosomal assemblies shown in green ( link B).

Each of the examples in the previous use cases corresponds to searches using the taxon index. Similar queries may also be made using the assembly index with the key difference being that the results will contain one row per assembly rather than per taxon. A limitation of the taxon index is that values may be aggregated across multiple assemblies such that in a plot of contig N50 and scaffold N50 a taxon may be represented by values from different assemblies. Using the assembly index allows comparison of contig and scaffold N50s for each assembly in the DToL BioProject, highlighting that the majority fulfil the EBP criterion of contig N50 > 1Mb and scaffold N50 > 10Mb ( Figure 16). The interactive nature of the reports allows outliers to be identified by clicking on the grid cells to refine the search. For example the sole haploid assembly with contig N50 below 200kb is *Senecio squalidus* (Oxford ragwort) assembly GCA_910822075.1.

### Data exploration

The versatility with which combinations of metadata can be queried in GoaT makes it a powerful resource to explore and discover biological patterns across the tree of life. The ability to generate reports directly from a query using the GoaT UI allows rapid visualisation of potential trends and can be used to identify the data to download for further analysis and visualisation in any external software. Cases 11 and 12 provide examples of basic data exploration to explore large-scale trends using the data in GoaT.

**Case 11 - Can plant plastid and mitochondrial assemblies be separated on the basis of GC content and span?**

**Figure 17.**
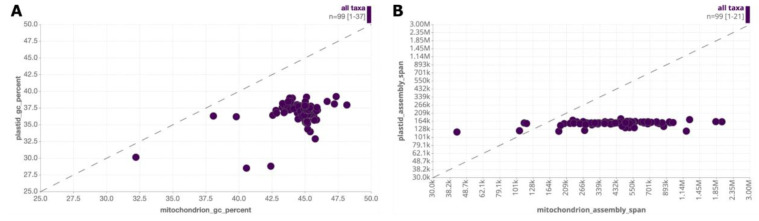
Exploration of plastid and mitochondrial genome characteristics. Scatter reports showing relationships between ( **A**) GC content ( Link A) and ( **B**) assembly span ( link B) for plant mitochondrial and plastid genome assemblies for all 99 Viridiplantae species in INSDC that have both organellar genomes present.

Plotting GC content of plastids versus mitochondria from plant (Viridiplantae) species shows that they have comparable variation, but plastid GC content is always lower than mitochondrial GC content for a given taxon ( Figure 17A). For assembly span, mitochondrial values are much more variable than plastid values ( Figure 17B). Four taxa have a greater plastid assembly span than mitochondrial assembly span, but the majority of taxa have a mitochondrial assembly span greater than the highest plastid value (176kb).

**Case 12 - Is there a correlation between the length of assemblies and gene content?**

**Figure 18.**
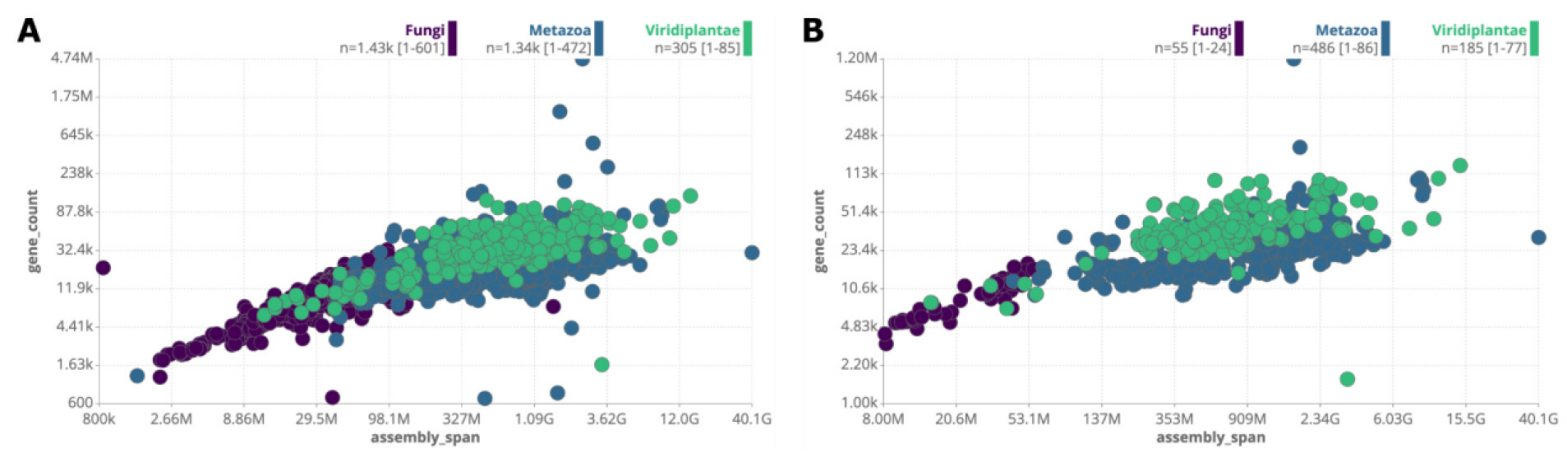
Scatter reports showing the relationship between assembly span and gene count by kingdom. ( **A**) all 3,078 assemblies ( Link A) and ( **B**) only 726 chromosome-level assemblies ( Link B).

Genome size is known to be influenced by factors such as ploidy, repeat content, and intron length ^26^. Very small genomes may also have reduced gene number ^27^. To assess the extent to which there is a more general relationship between genome size and gene number in assembled genomes, a report can be generated in GoaT of the relationship between *assembly_span* and *gene_count* ( Figure 18). All three eukaryotic kingdoms show a positive correlation across all assemblies ( Figure 18A). However, there are several outliers, including two assemblies with over 2 million predicted genes. Further filters can be applied to restrict the comparison to only chromosomal assemblies where the same trends remain apparent ( Figure 18B).

## Discussion

We have developed GoaT to meet the growing need for a flexible and scalable platform to help coordinate the efforts of Earth BioGenome Project affiliates to sequence all described eukaryotic species. GoaT functions as a search engine for project planning and coordination and provides tools for reporting and data exploration. The use of an Elasticsearch-based architecture helps to ensure that GoaT can scale to meet the ambitions of the Earth BioGenome Project. By including estimated values derived from descendant or sibling taxa, GoaT enables searching both directly recorded values as well as estimates. It also provides a demonstration of the scalability necessary to function across all eukaryotic species as GoaT already indexes attributes across over 1.8 million taxa.

GoaT maintains currency as projects progress and new data are published through a frequent update schedule. Core information derived from publicly available (INSDC) assemblies and updates to the underlying taxonomy (including new taxon registrations) are updated every weekday, while target and status lists supplied by EBP-affiliate projects are included in the next daily release after they have been pushed to the goat-data GitHub repository. This enables GoaT to be integrated into pipelines, such as the DToL production pipeline which uses the latest available estimated genome sizes to plan sequencing effort for each species, and the Arthropoda Assembly Assessment Catalogue ^28^ at https://evofunvm.dcsr.unil.ch/upcoming_assemblies.html, that uses information about sequencing status. Frequent updates of status lists, in particular, are fundamental to GoaT achieving its objective of helping to reduce duplication of sequencing efforts as the number and pace of large-scale sequencing projects continue to increase.

All searches in GoaT can be visualised in a range of graphical reports. These allow a rapid overview of data in GoaT and afford greater computational efficiency than exporting the raw data to produce the equivalent plots elsewhere. Nevertheless, all raw data for the reports in GoaT are available for download to enable users to perform initial data exploration within GoaT before exporting the data to an alternate platform where they can have complete control over data visualisation. The frequent update schedule ensures that GoaT visualisations provide live summaries of the available data that can be used for data exploration and project reporting. For the 25 EBP-affiliate projects with dedicated project pages on GoaT, these reports are used to provide a consistent snapshot of progress towards project goals. Each report is reproducible from the URL and can be embedded into external web pages so this live reporting functionality is accessible to GoaT users beyond these projects.

While we have described use cases that explore the data in GoaT to summarise projects or to explore biological patterns, GoaT reports also offer a visual depiction of outliers in the data returned by a query. In some cases these may be due to unusual biological characteristics, highlighting taxa that are perhaps less suitable for selection as initial targets for biodiversity genomics. Another class of outliers reveal errors in the data indexed by GoaT. These are values that should be excluded from analysis of trends across taxa and that ideally should be flagged as unreliable or removed from the index. We are currently developing a protocol for users to report values that they believe to be erroneous and are pursuing active curation of data sources to suppress such values more directly.

GoaT searches highlight the source of aggregated values, making it straightforward to use GoaT to identify clades with no descendant values. In the case of EBP-affiliated projects, this information for the assembly attributes is being used to direct sequencing efforts towards the least represented branches of the tree of life. For other attributes, it can highlight gaps in available data and serves as an indication of opportunities for targeted data curation to improve the availability of information on these attribute values for specific taxa. For example, while developing GoaT, we became aware that there was a paucity of karyotype data for the phylum Nematoda, prompting a targeted review of the relevant literature to collate this information (Blaxter *et al.*, in preparation) for inclusion in GoaT. Additional sources of karyotypic information absent from GoaT and uncovered by DToL assembly curators are currently under compilation and will be published as collated information in primary open-source data repositories.

GoaT has been implemented as an instance of the GenomeHubs platform, using the Configuration-as-Code paradigm to allow all GoaT-specific customisation to be defined through a set of YAML, TSV and Markdown files. This supports a highly flexible data model in which it is straightforward to index additional attributes to match changes in requirements and available data. The GenomeHubs codebase is under active development and we anticipate continuing to incorporate new developments into GoaT, for example to increase the scope of the sample index and to incorporate additional geographic data.

While the example use cases focussed on the DToL project, we hope that the rich insights that these use cases provide will encourage other sequencing projects to make their target lists and sequencing statuses available via regularly updated data dumps or APIs for GoaT to index. Target lists and sequencing status of any genome sequencing initiative can be included on GoaT by contacting GoaT curators at goat@genomehubs.org. A template file and guidelines can be found at https://goat.genomehubs.org/submissions. We also invite users and creators of other genome-relevant data to suggest new metadata sources, and to collaborate with us to build a comprehensive datastore and analytical platform that will power the Earth BioGenome Project's goal of sequencing all 1.9 million described eukaryotic species.

## Figures and Tables

**Table:** 

*Name*	*Email*	Conceptualization	Data Curation	Formal Analysis	Funding Acquisition	Investigation	Methodology	Project Administration	Resources	Software	Supervision	Validation	Visualization	Writing – Original Draft Preparation	Writing – Review & Editing
Richard Challis	rc28@sanger.ac.uk	X		X		X	X	X		X	X		X	X	X
Sujai Kumar	sk13@sanger.ac.uk		X	X				X	X	X		X		X	X
Cibele Sotero- Caio	cs43@sanger.ac.uk			X		X		X				X	X	X	X
Max Brown	mb39@sanger.ac.uk	X					X			X					X
Mark Blaxter	mb35@sanger.ac.uk	X			X						X				X

## Data Availability

All data and software versions described in this article are publicly available through open- source GitHub repositories and have been archived in the Zenodo open access repository. Zenodo: *genomehubs/genomehubs: 2.5.39*. https://doi.org/10.5281/zenodo.7324291. Contains the GenomeHubs codebase. From https://github.com/genomehubs/genomehubs. Software repository released under the MIT license. Zenodo: *genomehubs/goat-data: archive-2022.11.16*. https://doi.org/10.5281/zenodo.7331329. Contains all GoaT data and attribute metadata for release 2022.11.16 alongside scripts used during data preprocessing. From https://github.com/genomehubs/goat-data. Mixed configuration, data and software repository released under the MIT license. Zenodo: *genomehubs/goat-ui: archive-2022.11.16*. https://doi.org/10.5281/zenodo.7331372. Contains Markdown files and images used to apply GoaT-specific web User Interface customisation. From https://github.com/genomehubs/goat-ui. Configuration repository released under the MIT license. Zenodo: *genomehubs/goat-cli: Cypella*. https://doi.org/10.5281/zenodo.7348666. Contains version 0.2.5 of the GoaT CLI codebase. From https://github.com/genomehubs/goat-cli. Software repository released under the MIT license
