# Supplementary material for: Genomes on a Tree (GoaT): A versatile, scalable search engine for genomic and sequencing project metadata across the eukaryotic tree of life
Source: Wellcome Open Res. 2023 Jan 17;8:24. [Version 1] doi: 10.12688/wellcomeopenres.18658.1 (PMC9971660; doi:10.12688/wellcomeopenres.18658.1)
Supplement: Peer review history

# Peer review: Reviewer response for version 1

**DOI:** 10.21956/wellcomeopenres.20691.r54201

**Published:** 2023-02-01

**Authors:** Nuala O'leary

**Affiliations:** 1 National Center for Biotechnology Information, National Library of Medicine, National Institutes of Health, Bethesda, MD, USA

The Genomes of a Tree (GoaT) resource aims to provide the biology research community with access to enhanced genomic and project-level metadata for the vast array of eukaryotic genomic sequencing projects. As such, it is a valuable resource for any researcher or user looking for the current taxonomic scope, progress, and details of these sequencing projects. In addition, GoaT supports both web and programmatic users through its easy-to-use web interface, command line tool and API.

 The manuscript is well organized, and goals of the project are clearly defined. The methods, data models and data sources are presented with clarity and conciseness and accompanied by appropriate figures and tables to reinforce the value of the resource. The use of case studies is particularly beneficial for inexperienced users.

 The web interface is elegantly designed and easy to use.

 I recommend this paper for indexing with one minor point to address on the case studies. It would have been nice to see more detailed tutorials on how to use the command line (CLI) tool to address the case studies presented. As the GoaT documentation points out, APIs are complex and the CLI provides a more intuitive layer for users looking for programmatic access. Documentation on the CLI was a bit difficult to find.

Are the conclusions about the tool and its performance adequately supported by the findings presented in the article?

Yes

Is the rationale for developing the new software tool clearly explained?

Yes

Is the description of the software tool technically sound?

Yes

Are sufficient details of the code, methods and analysis (if applicable) provided to allow replication of the software development and its use by others?

Yes

Is sufficient information provided to allow interpretation of the expected output datasets and any results generated using the tool?

Yes

Reviewer Expertise:

Public database management and access

I confirm that I have read this submission and believe that I have an appropriate level of expertise to confirm that it is of an acceptable scientific standard.

# Peer review: Reviewer response for version 1

**DOI:** 10.21956/wellcomeopenres.20691.r54202

**Published:** 2023-01-30

**Authors:** Robert Waterhouse

**Affiliations:** 1 Department of Ecology and Evolution, University of Lausanne, Lausanne, Switzerland; 2 SIB Swiss Institute of Bioinformatics, Vaud, Switzerland

Challis *et al.* present the genome metadata aggregator tool, Genomes on a Tree (GoaT). GoaT collects and collates genome-relevant metadata for eukaryotic species from a variety of sources. These include not only genome assembly metrics but also genome size estimates, modes of sexual reproduction, and karyotype information, even for species lacking genome assemblies. Importantly, using available data GoaT also provides taxonomically informed estimates for taxa for which metadata are currently missing. The technical descriptions of the methods and implementations are detailed and clear. The use cases presented in the results provide good examples of the practical utility of the tool with several different applications. GoaT represents an important resource for mapping and tracking global efforts to build genome resources for all eukaryotic life, greatly facilitating the coordination and planning efforts of the umbrella Earth BioGenome Project and indeed researchers and all biogenome projects worldwide. 

 Abstract:

- Define API.

Keywords:

- “Databasing” seems somewhat informal, and e.g. returns just 5 hits in PubMed from 2022, “metadata” might be more relevant. “Elasticsearch” is a similarly niche term. 

Introduction:

- genomesize.com is probably better referred to as the Animal Genome Size Database

Methods:

- NCBI – define.
- “into the backbone taxonomy” – because the previous sentence mentions the GBIF backbone taxonomy here it becomes unclear which taxonomy GoaT is inserting additional taxa into.
- YAML – define.
- Figure 1: the figure title mentions “curation process” but “curation” is not mentioned in the main text, only “import”, so this becomes somewhat confusing.
- Figure 5: define UI.
- “tax_name(synonym:Sophophora melanogaster” – missing closing parenthesis.
- Data fetching section – indicate at the start that these are examples representing the current implementation and that the live API documentation should be consulted for query building.
- "Earth BioGenomes Project" - Earth BioGenome Project. 

Results:

- “are labelled chromosomal or complete” - for the non-expert it could be helpful to add a sentence about what these labels mean.
- Table 3: first column is not needed.
- “if the genome metadata suggest they may be outliers” - the concept of “outlier” is not very well explained and would benefit from some explanations.
- Figure 7: the exclusion of PRJEB40665 is not explained.
- “Within the Arthropoda” - Within Arthropoda.
- Figure 10: “(B)by family” - (B) by family.
- Figure 13: “aids in resolution of assembly issues” - this might be obvious to those working with Hi-C data for assembly curation but to most it would need a brief explanation

Discussion:

- “maintains currency” - potentially confusing phrasing.

Are the conclusions about the tool and its performance adequately supported by the findings presented in the article?

Yes

Is the rationale for developing the new software tool clearly explained?

Yes

Is the description of the software tool technically sound?

Yes

Are sufficient details of the code, methods and analysis (if applicable) provided to allow replication of the software development and its use by others?

Yes

Is sufficient information provided to allow interpretation of the expected output datasets and any results generated using the tool?

Yes

Reviewer Expertise:

Arthropod evolutionary and functional genomics, bioinformatics and computational biology

I confirm that I have read this submission and believe that I have an appropriate level of expertise to confirm that it is of an acceptable scientific standard.
